# Human Population Genetic History and Evolutionary Dynamics on the Eastern Tibetan Plateau

**DOI:** 10.1093/molbev/msaf258

**Published:** 2025-11-17

**Authors:** Guanglin He, Shuhan Duan, Gang Chen, Chuan-Chao Wang, Haibing Yuan, Xiangping Li, Qiuxia Sun, Fengxiao Bu, Jing Cheng, Yu Lu, Chao Liu, Huijun Yuan, Mengge Wang

**Affiliations:** Department of Oto-Rhino-Laryngology & Institute of Rare Diseases, West China Hospital of Sichuan University, Sichuan University, Chengdu 610000, China; Center for Archaeological Science, Sichuan University, Chengdu 610000, China; Department of Oto-Rhino-Laryngology & Institute of Rare Diseases, West China Hospital of Sichuan University, Sichuan University, Chengdu 610000, China; Department of Forensic Medicine, College of Basic Medicine, Chongqing Medical University, Chongqing 400331, China; School of Basic Medical Sciences, North Sichuan Medical College, Nanchong 637100, China; Hunan Key Laboratory of Bioinformatics, School of Computer Science and Engineering, Central South University, Changsha 410075, China; Department of Anthropology and Ethnology, Institute of Anthropology, School of Sociology and Anthropology, Xiamen University, Xiamen 361005, China; Center for Archaeological Science, Sichuan University, Chengdu 610000, China; Department of Oto-Rhino-Laryngology & Institute of Rare Diseases, West China Hospital of Sichuan University, Sichuan University, Chengdu 610000, China; Department of Oto-Rhino-Laryngology & Institute of Rare Diseases, West China Hospital of Sichuan University, Sichuan University, Chengdu 610000, China; Center for Archaeological Science, Sichuan University, Chengdu 610000, China; Department of Oto-Rhino-Laryngology & Institute of Rare Diseases, West China Hospital of Sichuan University, Sichuan University, Chengdu 610000, China; Center for Archaeological Science, Sichuan University, Chengdu 610000, China; Department of Oto-Rhino-Laryngology & Institute of Rare Diseases, West China Hospital of Sichuan University, Sichuan University, Chengdu 610000, China; Center for Archaeological Science, Sichuan University, Chengdu 610000, China; Department of Oto-Rhino-Laryngology & Institute of Rare Diseases, West China Hospital of Sichuan University, Sichuan University, Chengdu 610000, China; Center for Archaeological Science, Sichuan University, Chengdu 610000, China; Anti-Drug Technology Center of Guangdong Province, Guangdong 510000, China; Department of Oto-Rhino-Laryngology & Institute of Rare Diseases, West China Hospital of Sichuan University, Sichuan University, Chengdu 610000, China; Center for Archaeological Science, Sichuan University, Chengdu 610000, China; Shanghai Key Laboratory of Gene Editing and Cell Therapy for Rare Diseases, Fudan University, Shanghai 200031, China; Department of Oto-Rhino-Laryngology & Institute of Rare Diseases, West China Hospital of Sichuan University, Sichuan University, Chengdu 610000, China; Center for Archaeological Science, Sichuan University, Chengdu 610000, China; Department of Forensic Medicine, College of Basic Medicine, Chongqing Medical University, Chongqing 400331, China

**Keywords:** whole-genome sequencing, Tibetan Plateau, evolutionary history, genetic admixture, Tibetan-Yi and Hexi corridors

## Abstract

The origins of Tibeto-Burman populations on the eastern Tibetan Plateau (TP), especially within the Tibetan-Yi Corridor, remain unresolved. We sequenced whole genomes of 293 individuals from 21 Tibeto-Burman-speaking groups and genotyped 799 individuals from 60 Sino-Tibetan-speaking groups to reconstruct regional population history. Our analyses reveal fine-scale substructure and extensive admixture along the underrepresented Tibetan-Yi and Hexi corridors, driven by gene flow from Eastern Eurasian rice/millet farmers and Western Eurasian steppe pastoralists. We estimate that Tibetans diverged from their common ancestors with Han Chinese in the early Neolithic (∼9.9 kya), followed by differentiation among Tibetan-Yi Corridor populations in the middle Neolithic (∼4.6 kya). These splits coincide with distinct cultural trajectories that produced a pronounced north–south genetic structure among Tibeto-Burman groups. QpAdm modeling indicates that northern Tibeto-Burman speakers derive most of their ancestry from Neolithic millet farmers. Along the Hexi Corridor, an essential axis of Eurasian connectivity, fine-scale analyses show a dominant legacy of millet-farming populations with additional ancestry from incoming Eurasian herders. Together, these findings clarify the settlement history of eastern TP populations and underscore the role of geographic and cultural corridors in structuring ancient intercontinental gene flow across Eurasia.

## Introduction

Archaeological findings indicate that modern humans first inhabited the Tibetan Plateau (TP) approximately 40,000 to 30,000 calibrated years before present (YBP), with the northeastern fringes of the TP (NETP) acting as vital migration corridors (Zhang, et al. 2018). Evidence of intermittent Paleolithic human activity has been documented at several sites, including Quesang (20,000 YBP or 8,400 to 7,400 YBP at 4,270 meters above sea level [masl]), Jiangxigou1 (14,600 YBP at ∼3,200 masl), Heimahe1 (13,100 YBP at ∼3,200 masl), Xidatan2 (9,200 YBP at ∼4,300 masl), and Yeniugou (7,500 YBP at 3,800 masl) (Meyer, et al. 2017). These sites, predominantly located in the NETP, span from 20,000 to 7,500 YBP during the Paleolithic period, transitioning to Neolithic forager or agricultural sites influenced by lowland East Asian cultures during the Neolithic and Bronze Ages (Chen, et al. 2015). Recent research has indirectly characterized archaic humans in high-altitude East Asia through the Denisovan-inherited endothelial PAS domain protein 1 (*EPAS1*) gene, which is supported by an amino acid substitution found in a 160,000-year-old Denisovan fossil (Chen, et al. 2019) and Denisovan mitochondrial DNA from Baishiya Karst Cave (Zhang, et al. 2020). These findings suggest that early settlers undertook extensive exploration across high-elevation areas during the Paleolithic period, culminating in rapid, permanent settlements through the NETP during the Holocene, facilitated by novel agropastoral economies. Stylistic analyses of Holocene artifact assemblages indicate gradual cultural integration between the TP and North China, pointing to a potential single origin in North China and possible dispersal routes for Sino-Tibetan-speaking populations. Similarities between the Paleolithic microblade industries of the TP and southern Siberia further suggest a close connection between ancient TP populations and southern Siberians (Yue, et al. 2021). Additionally, ancient paternal lineages linked to D-M174 provide evidence of deep Paleolithic connections between Onge, Japanese, and Tibetan populations (Shi, et al. 2008). Despite these advances in the prehistory of TP regions, the genomic resources of geographically different Tibetan-Burman-speaking populations and deep demographic history have been underrepresented in human genetic and evolutionary research.

The Tibetan-Yi and Hexi corridors have been crucial in facilitating north-south and east-west cultural and genetic exchanges. Situated along the northeastern and eastern edges of the TP, these corridors, including an archaeological corridor in the NETP, have significantly contributed to the region's complex cultural, genetic, and ethno-linguistic diversity. The surrounding regions of the TP, predominantly inhabited by Altaic and Sino-Tibetan speakers, showcase a rich array of archaeologically documented Neolithic cultures and subsequent developments, spanning the Yangshao, Majiayao, Siba, and other periods through Bronze and Iron Ages to historical times (Chen, et al. 2015). Archaeological evidence indicates that, prior to 5,200 YBP, hunter-gatherers began migrating to higher elevations of the TP. This movement was later followed by settlers from the late Neolithic Yangshao culture, who adopted cold-tolerant barley agriculture and established permanent settlements in the TP's marginal regions. This transition marked the onset of the Ceramic Age in the Gansu-Qinghai region, characterized by a distinctive pottery style (Chen, et al. 2015). Two competing models have been proposed to explain the interactions between these newcomers and local populations. The demic diffusion model suggests that Yangshao farmers migrated southwestward from the middle Yellow River Basin (YRB) through the Gansu-Qinghai region to the Gonghe Basin, where they mixed with local hunter-gatherers. Conversely, the cultural diffusion model suggests that millet farmers who migrated to the Gonghe Basin intermittently exchanged material with local hunter-gatherers but did not significantly contribute to their gene pool (Chen, et al. 2015). Recent ancient DNA studies have shed light on interactions between Neolithic millet farmers and indigenous communities in the NETP Zongri area (Wang, et al. 2023). Nonetheless, the nature of these population interactions requires further exploration through comprehensive analyses of recent and ancient genome-wide DNA data, along with high-quality modern genomes, to comprehensively understand historical dynamics.

The peopling of the TP and the formation of modern Sino-Tibetan-speaking populations has been extensively studied across multiple research domains. Several ancient DNA studies have focused on the TP and surrounding lowland areas, whereas numerous modern genetic surveys have explored admixture processes involving both local populations and later migrants. Jeong et al. initially sequenced eight ancient genomes from the Himalayan arc, revealing a high-altitude East Asian origin and the long-term genetic stability of Himalayan highlanders (Jeong, et al. 2016). They later expanded their dataset to include 33 additional ancient genomes from the Himalayas, which primarily displayed farmer-related ancestry in the YRB alongside traces of deep Paleolithic Eurasian ancestry (Liu, et al. 2022). A comprehensive analysis of the genetic history of ancient humans on the TP revealed plateau-specific ancestry and significant genetic differentiation approximately 2,500 years ago. Subsequent gene flow from lowland East Asia has further shaped the genetic landscape of contemporary plateau populations (Wang, et al. 2023). A recent ancient DNA study of western TP populations highlighted the complex interconnections within and outside the TP (Bai, et al. 2024). In a separate study, Wang et al. analyzed 13 genomes from the Neolithic Wuzhuangguoliang site in Shaanxi Province and employed a qpGraph-based phylogenetic model to demonstrate that modern highland Tibetans derive approximately 84% of their ancestry from YRB farmers, with the remainder traced to ancient Eastern Eurasian hunter-gatherers, particularly the Onge (Wang, et al. 2021a). Ning et al. explored the ancient genetic diversity of Qijia farmers and Iron Age populations from Qinghai Province, revealing a close genetic relationship between the Qijia people and modern Tibetans (Ning, et al. 2020). Conversely, Ding et al. sequenced 67 complete mitochondrial DNA genomes from ancient Tibetans and identified two distinct population expansion events on the TP, challenging the idea of significant migration of lowland farmers to high-altitude areas (Ding, et al. 2020). Despite recent progress, genetic studies remain limited by limited sampling in the eastern and northeastern regions of the TP and adjacent corridors, hindering a comprehensive understanding of early peopling and fine-scale admixture dynamics. The genetic connections between temporally and geographically diverse ancient populations and present-day Sino-Tibetan groups also remain unresolved. Notably, demographic modeling using high-quality, representative genomes has revealed deep population histories in Oceania (Choin, et al. 2021), yet such approaches have been underutilized for East Asian populations. Consequently, the extent to which ancient TP inhabitants or northern Chinese millet farmers contributed to the gene pool of contemporary Sino-Tibetan-speaking populations remains unknown. These limitations underscore the need for expanded genome-wide data from peripheral regions to elucidate population origins and adaptive evolution in this area.

**Fig. 1.**
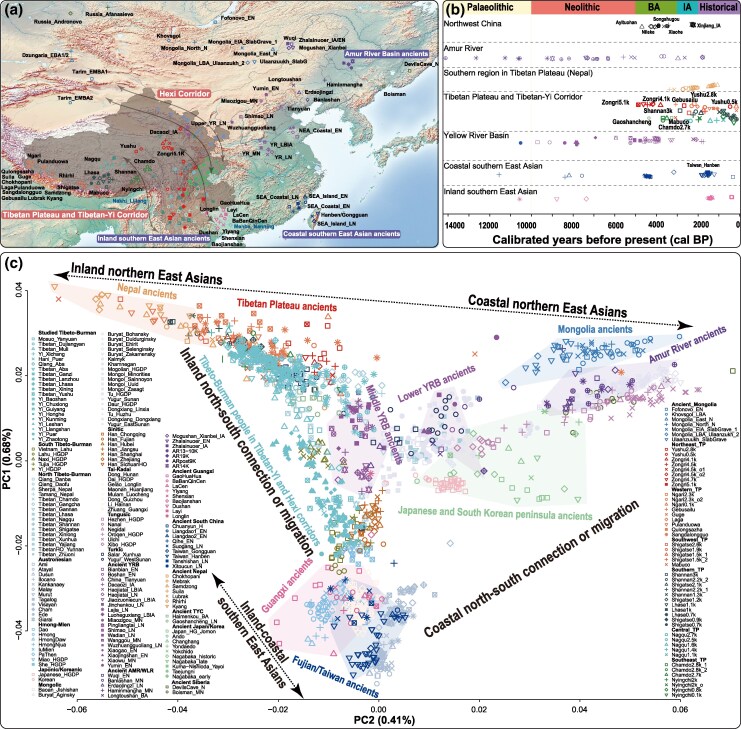
Genetic landscape of Sino-Tibetan-speaking populations in Eastern Eurasia. a) Geographic distribution of newly genotyped Sino-Tibetan groups alongside modern and ancient reference populations, providing a spatial context for the genetic background of the population. b) The temporal framework of ancient East Asian reference populations was plotted along a timeline of YBP. c) PCA of modern and ancient East Asians, in which ancient individuals were projected onto the patterns based on the two principal components estimated from modern population genetic variations. Modern individuals are grouped by language family in the PCA, with colored polygons indicating genetic or linguistically cultural affinities.

To elucidate the genetic diversity, origins, and demographic history of modern Sino-Tibetan-speaking populations along the Tibetan-Yi and Hexi corridors, we whole-genome sequenced 293 representative Tibeto-Burman-speaking individuals (Fig. 1a) and analyzed genome-wide data from 799 individuals across 60 geographically and ethnolinguistically diverse populations, along with deep genetic variation discovery and fine-scale demographic modeling. We incorporated all available ancient genomes from East Asia to model the demographic processes shaping the genetic landscape of modern Sino-Tibetan groups. Our study focused on key aspects of the genetic structure and admixture history of Sino-Tibetan groups. We first examined the genetic structure, relationships, ancestry composition, and potential genetic exchanges between Sino-Tibetan groups and their neighboring modern and ancient populations. We then explored the contributions of ancient farmers and pastoralists to the formation of modern Sino-Tibetan groups, identified the specific sources involved in their genomic admixture, and finally reconstructed the complex demographic history of these underrepresented populations. Our analysis revealed population substructures among modern Sino-Tibetan groups around the TP that aligned with linguistic affiliations and geographical divisions. We also observed additional gene flow from southern East Asians into populations in the Tibetan-Yi Corridor (TYC) and from Western herders into those in the Hexi Corridor, further complicating the genetic landscape of these regions. These findings provide a more nuanced understanding of the genetic underpinnings and historical interactions that have shaped the genomic diversity of contemporary Sino-Tibetan-speaking populations.

## Results

### Overview of Integrated Datasets and Genetic Variations

We generated WGS data for 293 Tibeto-Burman-speaking individuals from the Tibetan-Yi and Hexi corridors and surrounding regions (Fig. 1a; Table S1), with an average sequencing coverage of 40.7×. The resulting dataset comprised 20,537,967 single-nucleotide polymorphisms (SNPs) and 2,277,562 insertions and deletions (InDels) across autosomes, along with 808,176 SNPs and 99,498 InDels on the X chromosome. Among these variants, 7.7 million (32.7%) were singletons, 22.8% were rare (allele frequency [AF] < 0.01), and 14.8% were low in frequency (0.01 ≤ AF < 0.05). Notably, 1,693,241 SNPs (8.2%) and 165,261 InDels (7.3%) across autosomes were novel and absent from dbSNP v156, the 1,000 Genomes Project (1KGP), and the Westlake BioBank for Chinese (1000 Genomes Project et al. 2015; Cong, et al. 2022). Among the novel variants, 79.8% were singletons, and 18.5% were rare. In total, 18,737,137 biallelic autosomal SNPs were retained for downstream analyses. To comprehensively investigate the demographic history of the Tibetan-Yi and Hexi Corridor populations, an additional 799 Sino-Tibetan-speaking individuals from 60 populations were genotyped via high-density SNP microarrays, including 46 whole-genome sequenced samples (Fig. 1a; Table S1). These data were integrated with modern and ancient DNA datasets spanning diverse Eurasian populations, comprising 1,372 whole-genome sequenced individuals from 80 populations (Bergström et al. 2020; Choin, et al. 2021; He, et al. 2025), 2,298 Chinese individuals from 188 populations genotyped with the Affymetrix Array, 1,234 individuals from 139 modern and ancient populations in the 1240K dataset, and 1,885 individuals from 231 populations in the Human Origins (HO) dataset (table S1, Material online). We applied multiple analytical methods, including principal component analysis (PCA), ADMIXTURE, *f*-statistics, MSMC2, SMC++, fastsimcoal2, and fineSTRUCTURE, to explore fine-scale genetic structures, trace gene flow events, and delineate admixture and divergence patterns across these populations. These datasets and corresponding analyses provide a comprehensive view of the genetic landscape of the Tibetan-Yi and Hexi corridors, shedding light on the complex historical interactions and migration patterns that have shaped these regions.

### Genetic Clustering of Populations of Tibetan-Yi and Hexi Corridors

To investigate patterns of genetic relatedness between populations in the Tibetan-Yi and Hexi corridors and a broad range of Eurasian reference populations, we performed a series of PCAs using the expanded HO dataset. In the Eurasian-scale PCA based on the HO_Affy_WGS dataset, the studied population aligned along the central axis of the north-south genetic cline in East Eurasia. Highland Tibetans from Southwest China formed one end of this cline, trending toward Tungusic groups and ancient populations from the West Liao River and Amur River (AR) Basins. In contrast, other Sino-Tibetan-speaking groups shifted toward non-Sinitic modern populations and ancient individuals from southern East Asia and Southeast Asia (Figure S1). Both the newly genotyped Tibeto-Burman and Sinitic groups followed discernible clines; however, Han Chinese populations from the Tibetan-Yi and Hexi corridors clustered more tightly, showing stronger genetic affinities with non-Tibetan groups from the TYC (Figure S1b and c). The Eurasian-scale PCA based on the higher-density HO_WGS dataset reproduced the clustering pattern observed among the Tibeto-Burman groups in the HO_Affy_WGS dataset (Figure S2). To further refine the genetic structure of the Tibeto-Burman groups from the Tibetan-Yi and Hexi corridors, we conducted East Eurasia-specific PCA using the HO_WGS dataset. In this analysis, highland Tibeto-Burman-speaking populations and ancient individuals from the inland areas of the TP clustered at the western end of the northern East Asian cline, whereas coastal lowland Tungusic groups and ancient populations from the Mongolian Plateau (MP) and AR occupied the eastern end. In contrast, inland Tai-Kadai and Hmong-Mien groups, coastal indigenous Taiwanese, and southern East Asian ancient individuals clustered at the southern end (Fig. 1c). These findings revealed both inland-coastal and north-south genetic clines among East Asians, which is consistent with previously reported ancient genetic links between northern and southern East Asian populations, as well as between inland and coastal northern East Asian populations (Yang, et al. 2020; Wang, et al. 2021a; Liu, et al. 2025). Tibeto-Burman-speaking populations from the Tibetan-Yi and Hexi corridors formed a distinct genetic cline, bridging highland East Asians and lowland southern East Asians (Fig. 1c). Specifically, highland Tibeto-Burman groups in Southwest China showed strong genetic affinity with present-day Tibeto-Burman-speaking populations and ancient individuals from the TP, excluding those from the NETP; Tibeto-Burman groups in Northwest China were closely related to other linguistically similar groups from neighboring regions and ancient populations from the YRB and northeastern and southeastern TP; other Tibeto-Burman groups (Mosuo, Hani, Yi, and Qiang) in Southwest China clustered between highland Tibeto-Burman people and southern Han Chinese, overlapping with low- to mid-altitude Tibeto-Burman speakers from Southwest China and Southeast Asia, and displaying strong affinities with middle YRB ancient individuals (Fig. 1c). Overall, this PCA-based genetic structure highlights the complex and stratified genetic landscape of Tibeto-Burman-speaking populations in the Tibetan-Yi and Hexi corridors, shaped by geographic, cultural, and historical factors.

**Fig. 2.**
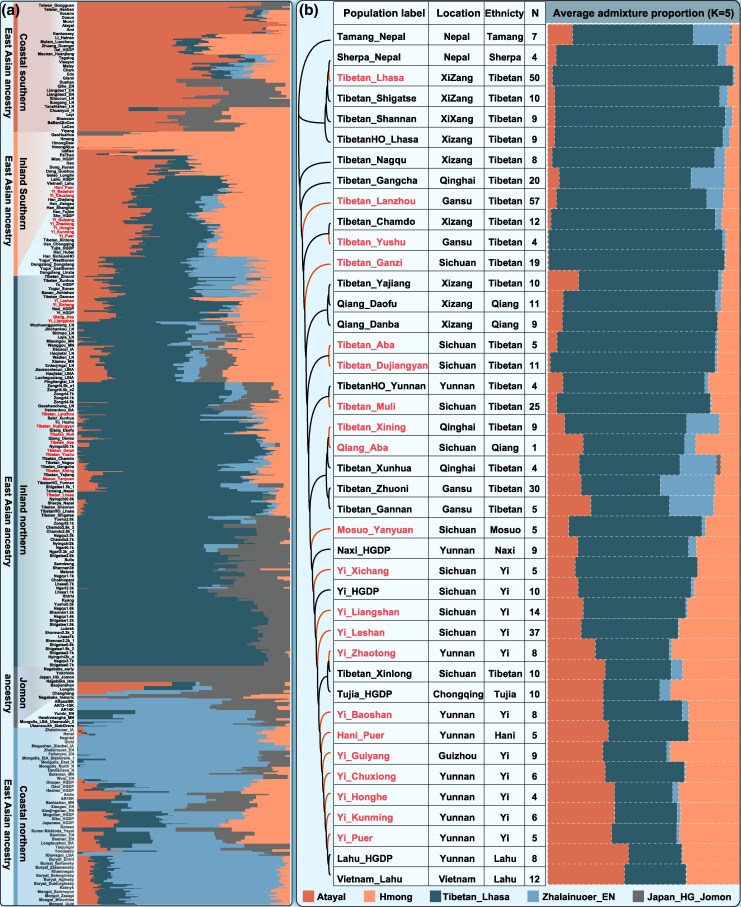
Population structure of Tibeto-Burman groups in the Tibetan-Yi and Hexi corridors inferred from the HO_WGS dataset. a) Model-based ADMIXTURE analysis of ancient and modern Eastern Eurasian groups, using five predefined ancestral components with the least cross-validation errors. The populations included are consistent with those shown in Fig. 1c. b) Genetic structure and clustering patterns of Tibeto-Burman groups from East and Southeast Asia. The left panel shows the phylogenetic relationships reconstructed from 1-outgroup-*f_3_* values, whereas the right panel displays the admixture profiles of the target Tibeto-Burman groups. Population labels, geographical regions, ethnicity, and sample size were presented between the tree and admixture results.

Model-based ADMIXTURE analysis of the HO_WGS dataset identified five major ancestral components among modern and ancient East Asian populations (Fig. 2). Two northern components corresponding to highland Tibetan and ancient Northeast Asian (ANA) ancestries predominated in populations from the TP and AR/MP regions; two southern components were enriched in Hmong-Mien- and Austronesian-related populations; and a distinct component was dominant among ancient individuals from Japan and South Korea. Tibeto-Burman groups from the Tibetan-Yi and Hexi corridors, except for Yi populations from Kunming (Yunnan), Honghe (Yunnan), and Guizhou, exhibited predominant highland Tibetan-related ancestry, with additional contributions from Austronesian- and Hmong-Mien-like ancestries. These three specific Yi populations carried substantial highland Tibetan, Austronesian, and Hmong-Mien-related components, although a larger proportion was derived from southern ancestries. Notably, Tibetan populations from Xining and Lanzhou in Northwest China presented substantial ANA-related ancestry, a pattern similar to that observed among other ethnic minorities in the Hexi Corridor (Fig. 2a). The genetic affinity among the Tibeto-Burman groups in the Tibetan-Yi and Hexi corridors was further supported by reconstructed phylogenetic relationships, *F*_ST_ genetic distances, and TreeMix-inferred population split patterns (Fig. 2b; Tables S2 and S3 and Figure S3). These findings, together with affinity signals from pairwise qpWave analysis, revealed pronounced genetic substructure within the Tibeto-Burman groups: those in the northwest presented more ANA-like ancestry; the middle- and high-altitude groups in the TP and TYC (mainly in western Sichuan) presented substantial highland Tibetan-related ancestry; and the low-altitude groups in the TYC (primarily in Yunnan) presented notable proportions of southern East Asian ancestry (Figure S4).

### Population Stratification Among Sino-Tibetan Groups in the TYC

**Fig. 3.**
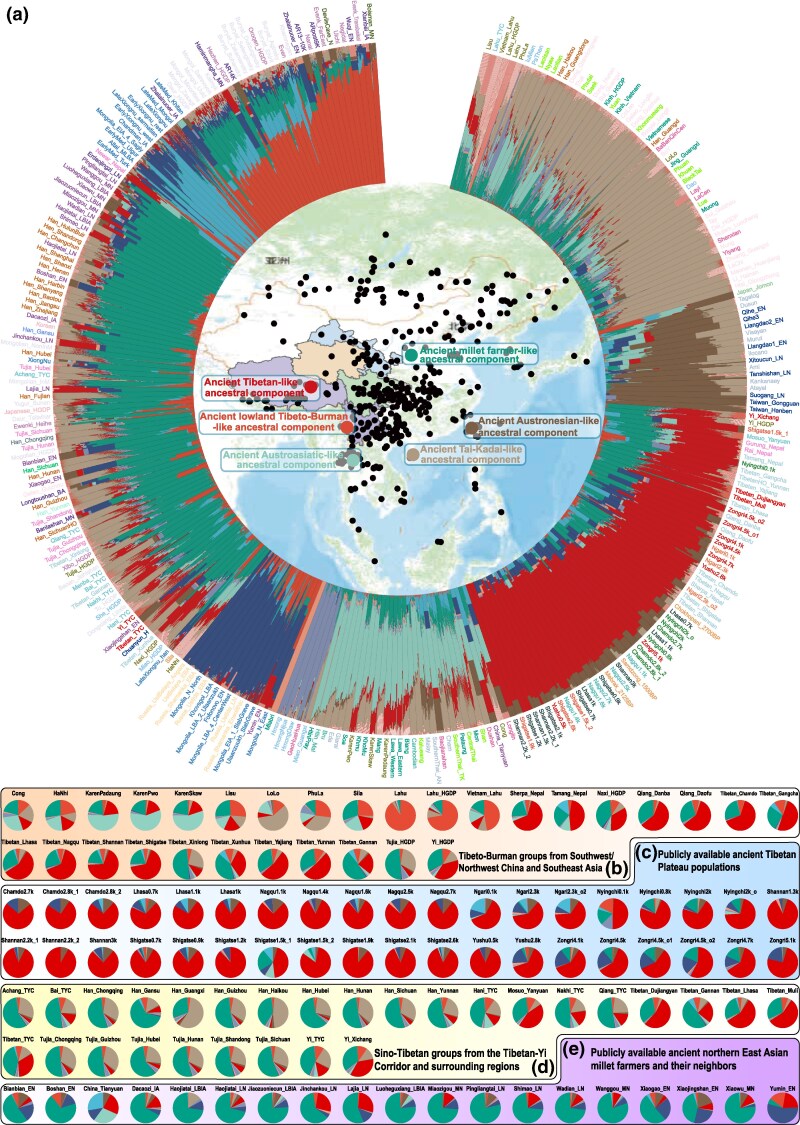
The admixture landscape of modern and ancient populations in Eastern Eurasia. (a) The ancestral composition of modern and ancient Eastern Eurasian populations at *K* = 11, as estimated from the HO_Affy dataset. The population groupings and color codes followed the scheme in Figure S1. The internal map of the ADMIXTURE analysis displays the geographic distribution of the populations included. The ancestral composition of Tibeto-Burman-speaking populations from southwest and northwest China, as well as Southeast Asia in the HO dataset b), ancient populations from the TP c), Sino-Tibetan groups from the TYC and surrounding regions, genotyped using the Affymetrix array d), and publicly available ancient northern East Asian millet farmers and neighboring ancient populations e).

**Fig. 4.**
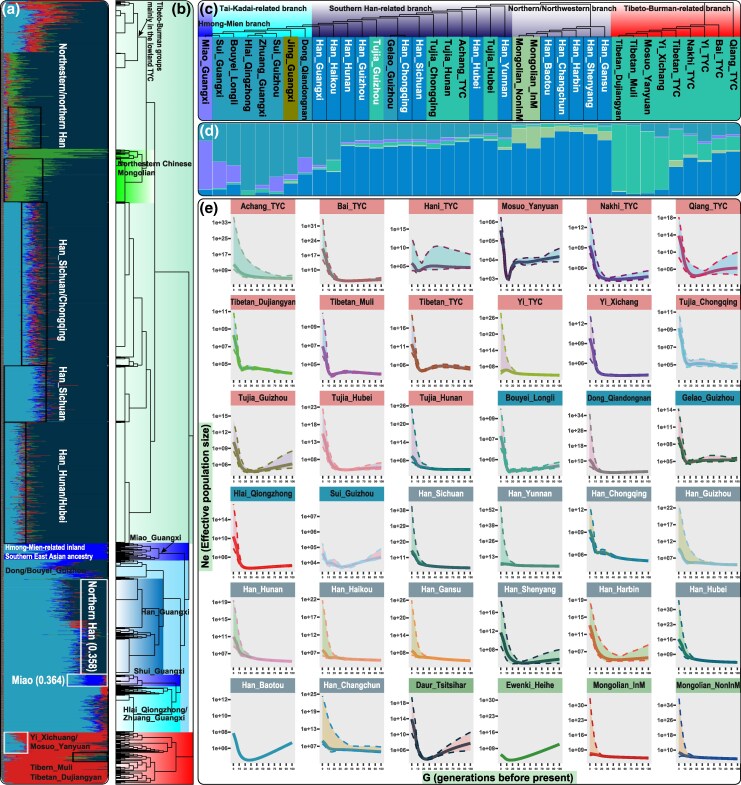
Fine-scale genetic structure of Sino-Tibetan-speaking populations in the Tibetan-Yi and Hexi corridors and neighboring groups inferred from the Affymetrix dataset. a) ADMIXTURE analysis of 2,736 East Asian individuals based on four predefined ancestral sources. b) Clustering patterns inferred from fineSTRUCTURE analysis of the pairwise coancestry matrix. c) Phylogenetic relationships among 36 East Asian groups reconstructed from the *F*_ST_ matrix. d) Ancestry composition bar plots showing mean ancestry proportions from the best-fit models among the Affy dataset, with geographically distinct but genetically/ethnically similar groups merged. e) Effective population sizes of the Sino-Tibetan-speaking and reference populations over the past 100 generations.

Populations within the Tibetan-Yi and Hexi corridors exhibited a distinct genetic cline linking the core TP with southwestern Chinese groups (Figure S1). To delineate the fine-scale population structure of Sino-Tibetan speakers in the TYC, we genotyped 799 individuals via a high-density Affymetrix array. PCA based on the HO_Affy dataset revealed clear stratification between northern Tibeto-Burman groups (primarily in China) and southern groups (mainly in Southeast Asia), along with notable differentiation among Sino-Tibetan-speaking populations within the corridor (Figure S5). Model-based ancestry inference confirmed these distinctions, identifying Lahu-specific and highland Tibetan-associated ancestries (Fig. 3; Figure S6). Highland Tibeto-Burman groups from the TP and western Sichuan harbored substantial highland Tibetan-like ancestry, peaking in ancient groups from the southwestern, southern, central, and southeastern TP. In contrast, other Sino-Tibetan groups within the corridor predominantly exhibited YRB-related ancestry, with additional southern East Asian components related to Tai-Kadai-speaking Hainan Li. Genetic differentiation among Sino-Tibetan groups in this region was further supported by outgroup-*f_3_* statistics, pairwise qpWave analyses and reconstructed phylogenetic relationships (Figures S7 to S9 and Tables S4 and S5). Observations from PCA and ADMIXTURE analyses at both East Asian and regional scales based on the high-density Affymetrix dataset also revealed distinct substructures within TYC populations (Fig. 4a; Figure S10a to d). The genetic cline observed here diverged from those in southern China, although lowland Tibeto-Burman groups within the corridor exhibited close affinities with lowland Sino-Tibetan groups in Southwest China (Figure S10a and c,). Admixture modeling identified a unique ancestral component in highland Tibeto-Burman groups such as Tibetan, Mosuo, and Yi, whereas other Sino-Tibetan groups derived their ancestry mainly from lowland northern and southern East Asian sources, with minor contributions from highland Tibeto-Burman-like ancestry (Fig. 4a; Figure S10b and d).

Shared genetic drift estimated by *f_4_* (Sino-Tibetan1, Sino-Tibetan2; Eurasian populations, Mbuti) revealed that lowland Tibeto-Burman and Sinitic groups in the TYC shared more alleles with southern East Asian Neolithic to historic populations and middle to upper YRB ancient populations than their highland counterparts did. In contrast, the highland Tibeto-Burman group exhibited greater drift with respect to the TP group than the lowland group did (Table S6). Within Tibetans, populations from central and southern Sichuan, such as Dujiangyan Tibetan and Muli Tibetan, shared more alleles with TP ancients than those from northwestern and western Sichuan. Haplotype-based tests further confirmed the fine-scale population substructure among Sino-Tibetan groups in the corridor (Fig. 4b; Figures S11 to S21). To formally characterize the admixture landscape of Sino-Tibetan groups in the TYC, we applied qpAdm-based two-way models using ancestral sources related to deeply diverged Neolithic YRB farmers and Neolithic southern East Asian or Southeast Asian hunter-gatherers and rice farmers. The estimates identified YRB farmers (represented by Miaozigou_MN) as the dominant ancestral source, contributing between 85.7% ± 5.6% and 92.6% ± 6.0% (Table S7). In three-way admixture models incorporating northern East Asian, coastal southern East Asian, and inland southern East Asian sources, highland Tibeto-Burman groups presented elevated YRB farmer-related ancestry compared with other Sino-Tibetan groups. In contrast, the lowland Sino-Tibetan group had a greater contribution from coastal southern East Asian ancestry, as represented by Ami and Taiwan_Hanben (Table S7 and Figure S10e and f). Additionally, Tibeto-Burman groups from the TP, western Sichuan, and Southeast Asia retained notable proportions of early Asian-related ancestry. The findings underscore the complex genetic interactions between major farming populations and local hunter-gatherer groups that shaped the demographic history of TYC populations. Clustering patterns, in which geographically distinct but genetically similar populations were merged, recapitulated premerger genetic structures (Fig. 4c and d). Effective population size estimates based on merged groups indicated that the lowland Hani, highland Mosuo, and Tibetan populations in the TYC experienced bottleneck events, whereas most other Sino-Tibetan groups exhibited expansions beginning approximately 10 to 20 generations ago (Fig. 4e). Lowland Yi exhibited a slight population decline within the past 10 generations; Sichuan Qiang and Guizhou Tujia showed reductions 20 to 25 generations ago, followed by subsequent expansions; and Yunnan Han displayed no significant change in population size. Admixture dating estimates indicated that the north-south genetic mixture in the Tibeto-Burman groups within the TYC likely occurred between 500 and 6,000 years ago (Table S8).

### Complex Deep Demographic Histories of TYC Populations

**Fig. 5.**
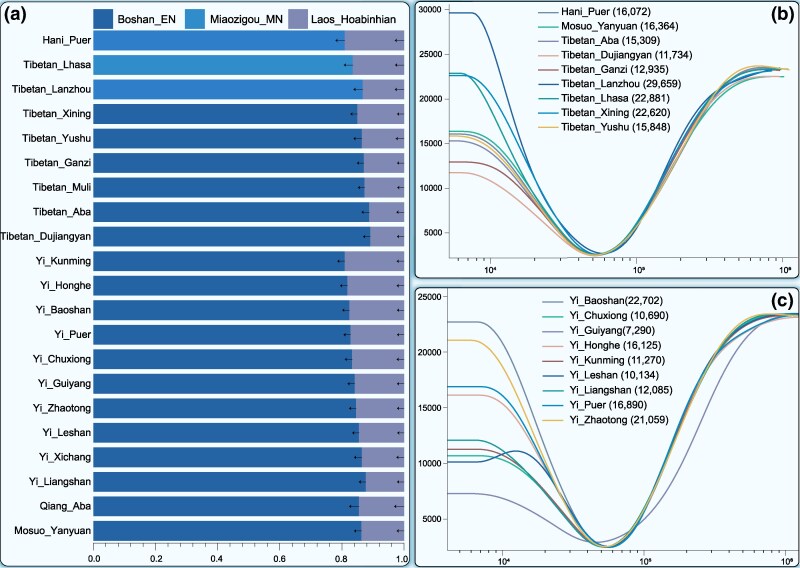
Formal admixture models and effective population size dynamics of whole-genome sequencing TYC populations. a) Formal admixture models inferred using qpAdm, showing ancestry proportions from deep northern and southern Chinese sources across TYC populations. Error bars represent standard errors of the admixture proportion estimates. b and c) Historical effective population size (*N*_e_) trajectories of Tibeto-Burman-speaking populations inferred using the SMC++ method. b) Temporal changes in effective population size among the Tibeto-Burman-speaking Hani, Mosuo, and Tibetan populations. c) Temporal changes in effective population size across geographically distinct Yi populations.

Clustering patterns inferred from whole-genome sequenced Tibeto-Burman groups closely mirrored those derived from genotyped datasets, enabling reconstruction of the complex demographic history of TYC populations via the expanded WGS dataset. We employed two-way distant qpAdm admixture models, which have recently been used to model the genomic formation of ancient and modern Tibetans (Liu, et al. 2022; Wang, et al. 2025), to explore the genomic composition of TYC populations. The model used Boshan_EN as a proxy for northern ancestry and Hoabinhian for southern ancestry, effectively capturing the genetic profiles of most Tibeto-Burman groups in the region. Compared with their lowland counterparts, the highland groups, excluding Aba Qiang, presented increased YRB-related ancestry (∼86% to 89%) (Fig. 5a). An alternative two-way model using Miaozigou_MN and Hoabinhian fit the genetic profile of the lowland Pu’er Hani. Estimates of the effective population size revealed that the Tibeto-Burman-speaking Hani, Mosuo, and Tibetan groups in the TYC generally maintained smaller effective population sizes than did the Tibetan groups in the TP and Northwest China (Fig. 5b). Demographic trajectories varied across geographically distinct Yi populations: those in the TYC began expanding ∼50,000 years ago; Leshan Yi experienced growth between ∼50,000 to 15,000 years ago, followed by a decline; and Guizhou Yi showed modest growth ∼40,000 years ago (Fig. 5c).

**Fig. 6.**
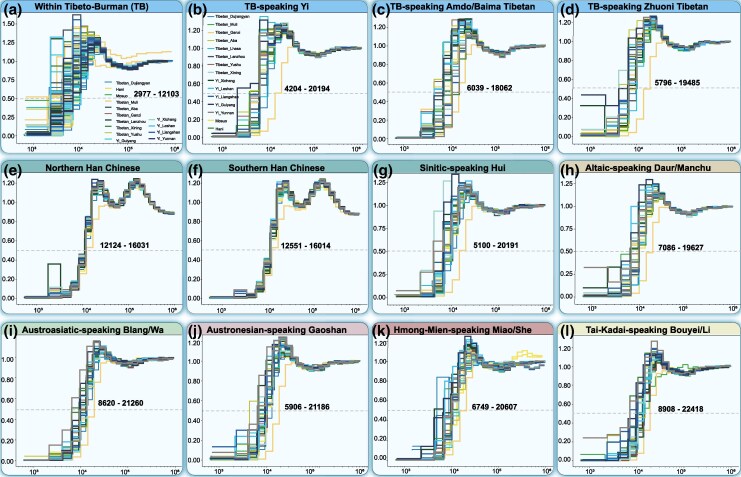
Complex demographic histories of Tibeto-Burman-speaking populations in the Tibetan-Yi and Hexi corridors. a) Intragroup divergence times within newly studied Tibeto-Burman populations. b to d) Divergence times between focal Tibeto-Burman groups and reference Tibeto-Burman-speaking populations: southern Tibeto-Burman-speaking Anha Yi from Sichuan b), Amdo Tibetan and Baima Tibetan c), and northern Tibeto-Burman-speaking Zhuoni Tibetan from Gansu d); e to g) divergence times estimated between TB groups and Sinitic-speaking populations from northern Chinese Han (CHB, e), southern Han Chinese (CHS, f), and Sichuan Hui g); h to l) Divergence times between TB people and linguistically diverse non-Sino-Tibetan speakers: Altaic-speaking Daur and Manchu from Inner Mongolia h), Austroasiatic-speaking Blang and Wa from Yunnan i), Austronesian-speaking Gaoshan from Fujian and Taiwan j), Hmong-Mien-speaking Sichuan Miao and Fujian She k), and Tai-Kadai-speaking Guizhou Bouyei and Hainan Li l).

We further investigated divergence patterns among Tibeto-Burman-speaking populations and between these groups and linguistically distinct northern and southern East Asians. Divergence time estimates revealed that southern Tibeto-Burman groups began splitting ∼3.0 to 20.2 kilo years ago (kya), and the divergence between focal and northern Tibeto-Burman groups occurred ∼5.8 to 19.5 kya (Fig. 6a to d; Figure S22a to d). We also estimated the divergence between Tibeto-Burman groups and northern or southern Han Chinese at ∼12.1 to 16.0 kya, with Sinitic-speaking Hui at ∼5.1 to 20.2 kya and with Altaic-speaking Daur and Manchu at ∼7.1 to 19.6 kya (Fig. 6e to h; Figure S22e to h). Focusing on the divergence from southern East Asian populations, we found that Tibeto-Burman groups in the TYC split from Austroasiatic-speaking Blang and Wa at ∼8.6 to 21.3 kya; Austronesian-speaking Gaoshan from Fujian and Taiwan at ∼5.9 to 21.2 kya; Hmong-Mien-speaking Sichuan Miao and Fujian She at ∼6.7 to 20.6 kya; and Tai-Kadai-speaking Hainan Li and Guizhou Bouyei at ∼8.9 to 22.4 kya (Fig. 6i to l; Figure S22i to l). These results underscore the complex demographic history of Tibeto-Burman-speaking populations in the TYC, shaped by both geographic isolation and admixture.

**Fig. 7.**
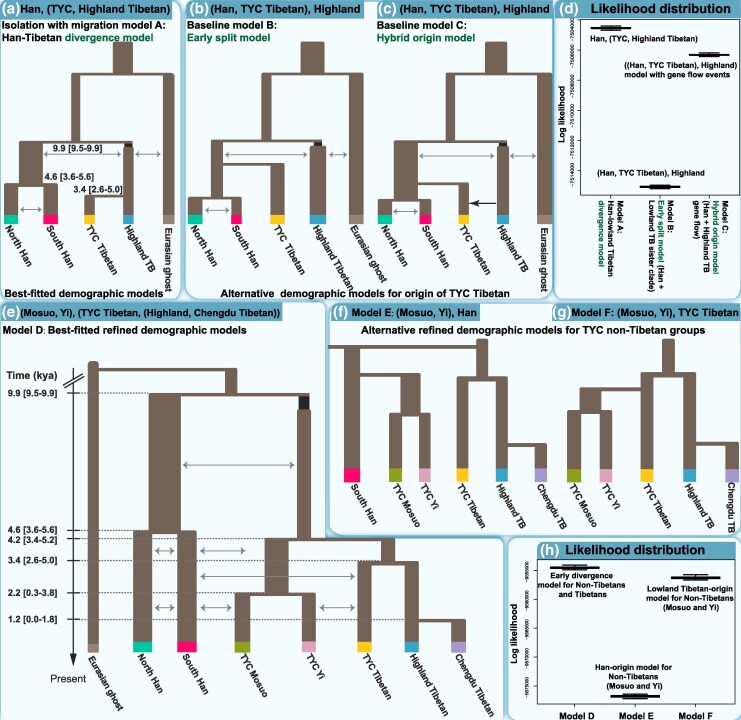
Complex demographic models of the peopling history of the TYC. a) ML baseline Han–Tibetan divergence model for the Sino-Tibetan-speaking Tibetan group from the TYC. b and c) Alternative topologies derived from the baseline model. b) An early divergence model in which Han and lowland Tibetans form a sister clade and c) a hybrid origin model involving gene flow from highland Tibetans into lowland Tibetans. The gray arrows indicate continuous gene flow (bidirectional arrows denote symmetrical flow). In contrast, the black arrows represent gene flow from highland Tibetans (Tibetan from Lhasa) to lowland Tibetans (Tibetan from Muli). The black rectangles represent population bottlenecks, with widths proportional to the estimated effective population size. d) Likelihood distributions for the three baseline models. Lines, boxes, whiskers, and dots indicate the median, interquartile range (IQR), 1.5×IQR, and outliers, respectively, based on 100 expected site frequency spectra calculated with 10^7^ coalescent simulations and using the same parameters that maximized the likelihood of each scenario. e) Best-fitting demographic model for TYC populations. The 95% CIs are displayed in parentheses. The analyses assume a mutation rate of 1.25 × 10^−8^ mutations per site per generation and a generation time of 29 years. The full parameter estimates and 95% CIs are provided in Table S9. f and g) Schematic representations of two refined alternative models for TYC populations; for clarity, only TYC populations and a single Han Chinese group are shown. h) Likelihood distributions for the best-fitting model in Fig. 6e and the two alternatives in Fig. 6f and g.

The genetic heterogeneity observed among linguistically distinct population pairs and the limited resolution of Holocene events prompted a formal reconstruction of evolutionary models for Tibeto-Burman populations using the site frequency spectrum. Thus, to determine the relationship between highland and lowland Tibetans, we used coalescence and composite likelihood methods to explore three linguistically and evolutionarily supported hypotheses for the origin of Tibetans from the TYC, including isolation with the migration model of the Han-Tibetan divergence model, early split from the highland Tibetans model and hybrid origin among the Han Chinese and Tibetan models. We first modeled population divergence among Sino-Tibetan-speaking populations, including Muli Tibetan in the TYC, Lhasa Tibetan in the TP, and Han Chinese groups (Northern Han and Southern Han) (Fig. 7a to c), and supported the early Neolithic divergence of Tibetan and Han Chinese and the common origin of geographically different Tibetans (Fig. 7d). We next expanded the model to include three additional ethnically diverse Tibeto-Burman-speaking populations, Xichang Yi and Yanyuan Mosuo (TYC) and Dujiangyan Tibetan (peripheral to the corridor), and explored the evolutionary patterns within the Tibetan-Burman people (Fig. 7e to h; Table S9). The optimal model supported the common origin among Tibetans and other Tibeto-Burman-speaking populations and dated the split between the Tibetan and non-Tibetan Tibeto-Burman groups to ∼4.2 kya (95% confidence interval [CI]: 3.4 to 5.2 kya), preceded by a significant population bottleneck (Fig. 7e and h). Muli Tibetans diverged from highland Tibetan groups ∼3.4 kya (95% CI: 2.6 to 5.0 kya), and Dujiangyan Tibetans separated from Lhasa Tibetans ∼1.2 kya (95% CI: 0.0 to 1.8 kya). Additionally, Xichang Yi and Yanyuan Mosuo split ∼2.2 kya (95% CI: 0.3 to 3.8 kya). Together, these findings suggest that the Tibeto-Burman-related ancestry observed across the core TP and eastern TP corridor regions derives from proto-Tibeto-Burman populations associated with Neolithic cultural expansion and dispersal.

### Western Eurasian Admixture in Hexi Corridor Populations

The Hexi Corridor, which is situated in the northern NETP and connects the western and central Eurasian steppe zones, has historically served as a key conduit for trans-Eurasian cultural exchange, human mobility, and admixture. To evaluate the extent of Western Eurasian ancestry in modern northwestern Chinese populations, we genotyped contemporary groups from the Hexi Corridor and adjacent regions, integrating these with geographically proximate but ethnolinguistically diverse populations. Clustering patterns revealed by PCA based on the HO_Affy_WGS and HO_WGS datasets revealed that the Tibeto-Burman groups from the Hexi Corridor formed a distinct genetic cline, separating them from those in the TYC (Fig. 1c; Figures S1 and S2). Han Chinese individuals from the Hexi Corridor also deviated from the genetic clines formed by Tibeto-Burman groups in both corridors and from southern Han Chinese individuals (Figures S5 and S10c). Ancestry deconvolution indicated that Tibetan groups in and around the Hexi Corridor, excluding Gangcha Tibetan, primarily derived their ancestry from YRB-related sources, with additional contributions from highland Tibetan, southern East Asian, ANA, and western steppe ancestries (represented by lateXiongnu_Sarmatian) (Fig. 3; Figure S6). Han Chinese in the region presented similar ancestry profiles but elevated YRB-related components, followed by southern East Asian ancestry and minor contributions from highland Tibetan, ANA, and western steppe lineages. Genetic differentiation among Sino-Tibetan groups in the Hexi Corridor and neighboring regions, as well as between northwestern and southwestern Tibeto-Burman groups, was corroborated by patterns of *F*_ST_ genetic distances, outgroup-*f_3_* statistics, pairwise qpWave tests, and phylogenetic reconstructions (Tables S2 to S5 and Figures S7 to S9).

To investigate the potential genetic influence of Western Eurasia on northwestern Chinese populations, we conducted a TreeMix analysis that consisted of modern Western and Eastern Eurasians with various gene flow events. This analysis detected gene flow from Western Eurasian admixtures related to French populations into the Gannan Tibetan and Gangcha Tibetan groups (Figure S3). To further characterize the impact of Neolithic-to-historic Western Eurasian gene flow and trans-Eurasian cultural interactions, we systematically investigated Western Eurasian ancestry in modern northwestern Chinese populations via affinity and asymmetrical *f*_4_-statistics. The estimates from *f_4_* (Southwest Sino-Tibetan, Northwest Sino-Tibetan; Eurasian populations, Mbuti) indicated that northwestern Sino-Tibetan groups shared more alleles with ANA and Western Eurasian ancestry than their southwestern counterparts did (Table S6). Additional comparisons using *f_4_* (northwestern Chinese1, northwestern Chinese2; Western Eurasians, Mbuti) revealed that ethnic minorities in Northwest China shared more genetic drift with Western Eurasians than nearby Han Chinese did, with the most drift observed in linguistically divergent ethnic minorities relative to northwestern Tibetans (Table S10).

We confirmed the presence of Western Eurasian ancestry across northwestern populations using qpAdm analysis (Table S11). Two-way admixture models with northern and southern East Asian proxies and three-way models incorporating PCA-extreme sources consistently failed to fit northwestern groups without including Western Eurasian ancestry (Table S7). Three- and four-way admixture models incorporating Western Eurasian populations such as Iran_C_SehGabi, Iran_BA1_ShahrISokhta, Pakistan_IA_Loebanr, Pakistan_IA_Katelai (southern farmers), and Alan, Sarmatian, Kangju, Afanasievo, Andronovo, Sintashta, and TianShanHun (northern pastoralists) successfully modeled most northwestern Chinese and southern Siberian groups but not Uyghurs and Kazakhs (Figure S23 and Table S11). These latter populations required three-way models with two Western sources and one Eastern source. For northwestern Tibetans, a three-way admixture model comprising Lajia_LN (80.7% to 86.3%), Taiwan_Hanben (6.2% to 13.8%), and Russia_Sarmatian (4.5% to 7.6%) provided the best fit. Northwestern Han Chinese were well modeled by Pingliangtai_LN (74.6% to 92.6%), Taiwan_Hanben (4.2% to 24.4%), and Russia_Sarmatian (1.0% to 4.5%), with a decreasing west-to-east gradient in the Sarmatian component. We also observed clear differences in ancestry composition between the Hexi Corridor and southern Siberian populations (Figure S23). Finally, four-way admixture models for populations in and around the Hexi Corridor suggested contributions from four major sources—northern and southern East Asians, as well as northern and southern Western Eurasians—shaping the genetic structure of present-day populations in this region (Table S11). This pattern aligns with previous findings in Xinjiang Uyghurs, where East Asian, Siberian, South Asian, and Western Steppe ancestries contributed to their complex genetic landscape (Feng, et al. 2017; Zhang, et al. 2021; Kumar, et al. 2022). Admixture dating using ALDER indicated that east–west gene flow events occurred between 390 and 1,600 YBP (Table S8), which is consistent with the admixture times inferred from GLOBETROTTER and recent population genetic modeling from ethnic minority genomes and ancient DNA (Xiong, et al. 2024; He, et al. 2025).

## Discussion

### Integrative Genomic Insights into the Genetic Legacy of Eastern TP Populations

The Yellow and Yangtze River Basins in China, alongside Southwest Asia, represent early centers of agriculture, marked by the domestication of millet and rice, respectively (Zuo, et al. 2017; Ma, et al. 2020). However, the genetic impact of these agricultural transitions on Chinese populations remains insufficiently characterized. In contrast to early Near Eastern farmers, whose descendants from regions such as Anatolia, the Levant, and Iran remained genetically distinct from pre-Neolithic hunter-gatherers, East Asian agricultural development involved substantial admixture with indigenous populations (Lazaridis, et al. 2016). Extensive genetic studies of ancient Western Eurasian populations have demonstrated pervasive admixture events, reshaping the ancestry of groups across Europe, the Eastern steppe, South Asia, and East Africa (Lazaridis, et al. 2016). Recent genomic evidence indicates that northern East Asians began cultivating dryland crops such as foxtail and broomcorn millet as early as ten thousand years ago (Ning, et al. 2020). However, the extent to which these early agriculturalists contributed genetically to subsequent populations across the YRB, Hexi Corridor, and TYC remains unclear. Moreover, ancient DNA analyses revealed eastward migrations of steppe populations and barley cultivators associated with the Bactria–Margiana Archaeological Complex (BMAC), which significantly shaped the gene pool of populations in Xinjiang and the Eastern Eurasian steppe (Jeong, et al. 2020; Kumar, et al. 2022). The extent of this genetic influence on neighboring regions, particularly the Tibetan-Yi and Hexi corridors, has yet to be systematically assessed.

We reported WGS data from 293 modern Tibeto-Burman-speaking individuals, including well-known Tibetan and other under-representative Yi, Hani, Qiang, and Mosuo individuals, and genome-wide SNP data from 799 individuals across 60 ethnolinguistically diverse populations from the Hexi and Tibetan-Yi corridors, regions historically marked by migration and admixture. Through allele- and haplotype-based analyses, we dissected the fine-scale population substructures of contemporary Sino-Tibetan groups. Multiple analytical approaches consistently revealed that highland Tibeto-Burman-speaking populations shared strong genetic affinities with ancient TP individuals, whereas lowland Tibeto-Burman groups from the Tibetan-Yi and Hexi corridors were more closely related to ancient populations from the YRB. Our findings underscore the deep genetic continuity between Neolithic YRB millet farmers and both ancient and present-day populations on the TP and TYC. Moreover, genome-wide data from the Hexi Corridor highlighted the region's role in trans-Eurasian cultural and demographic exchanges. The populations in this study predominantly derived their ancestry from Yangshao-related millet farmers, with minor but detectable gene flow from Western Steppe or BMAC-related sources. Together, our models clarify the demographic impact of Neolithic millet agriculturalists on both Sino-Tibetan and non-Sino-Tibetan populations across the Tibetan-Yi and Hexi corridors. They also detail substantial Western Eurasian genetic contributions to the Hexi Corridor populations. This study advances our understanding of the peopling history of the Tibetan-Yi and Hexi corridors, which are shaped by sustained migration, admixture, and cultural interactions over millennia.

### Genetic Structure and Admixture Dynamics of Contemporary Sino-Tibetan-Speaking Populations

Genomic analyses indicate that modern East Asians descended from two deeply diverged ancestral lineages dating back to the early Neolithic period (Yang, et al. 2020). Archaeological evidence suggests that three major north-south corridors facilitated cultural and population exchanges between ancient South China and North China (He, et al. 2017). Recent ancient DNA studies have illuminated Holocene expansions and admixtures along the eastern coastal and central corridors (Yang, et al. 2020; He, et al. 2021). However, the admixture dynamics within the TYC remain underexplored. By genotyping and sequencing modern Sino-Tibetan-speaking individuals from the TYC, we revealed marked genetic differentiation not only between the Sinitic and Tibeto-Burman groups in the corridor but also between northern and southern Tibeto-Burman speakers. These patterns reflected complex admixture processes involving both inland and coastal southern East Asians, as well as northern sources from the YRB. These observations underscore a strong genetic link with Neolithic millet farmers, supporting a North China origin of Sino-Tibetan-speaking populations and their languages. Our findings challenge the western Sichuan origin hypothesis by showing dominant ancestry from ancient millet farmers, without evidence for alternative ancestral sources within TYC populations. Southern Tibeto-Burman groups, especially lowland ones, exhibited substantial genetic admixture with local Hmong-Mien, Tai-Kadai, Austronesian, and Austroasiatic populations, suggesting closer genetic ties to southern East Asians. The genetic profiles of the TYC populations reflected predominant YRB-related ancestry, complemented by a balanced contribution from inland and coastal southern East Asian sources. Our three-way admixture model supports the North China origin hypothesis and offers further resolution to long-standing debates on the origins of Tibeto-Burman speakers (Sagart, et al. 2019; Zhang, et al. 2019; Wang, et al. 2021a). The observed genetic structure not only aligns with archaeological evidence linking the southward expansion of ancient populations associated with Majiayao culture but also highlights the pivotal role of the Crescent-Shaped Cultural Communication Belt in shaping population dynamics in this region. Demographic modeling suggested that the divergence of Tibeto-Burman-speaking populations in Southwest China began in the late Neolithic. Southwestern Tibetan groups shared a common ancestor distinct from those of the Yi and Mosuo, who, in turn, traced their ancestry to a separate lineage not shared with the Tibetan populations within the TYC. Additionally, the lowland Chengdu Tibetan population shared a more recent common ancestor with highland Tibetans, diverging around ∼1.2 kya, likely due to a recent migration event from the TP to lowland areas. The reconstructed demographic models with gene flow suggested an early Neolithic common ancestor between the Han Chinese and Tibetan-Burman people and a middle Neolithic separation within the northern Tibetan-Burman people.

### Transcontinental Migration and Western Eurasian Genetic Impact in the Hexi Corridor

Historical migrations across Eurasia profoundly shaped the genetic landscape of Hexi Corridor populations, reflecting complex interactions between ancient agriculturalists and pastoralists in this strategic region. Serving as a major corridor for bidirectional human movement, the Hexi Corridor connects western Eurasia with East Asia. Recent studies have revealed intricate admixture patterns in Xinjiang, adjacent to the Hexi Corridor, especially during the Bronze Age (Feng, et al. 2017; Ning, et al. 2019; Wang, et al. 2021b; Kumar, et al. 2022; He, et al. 2025). Large-scale genomic analyses revealed substantial west–east gene flow during this period, with Central Asian-derived maternal lineages and affinities to steppe pastoralists (Wang, et al. 2021b). Autosomal evidence further supports extensive interactions between Western and Eastern Eurasian groups (Kumar, et al. 2022). Ning et al. and Pan et al. identified Yamnaya-related ancestry in early Iron Age Xinjiang populations and detected Western Eurasian genetic components in modern Xinjiang residents (Ning, et al. 2019; Pan, et al. 2022). Moreover, Zhang et al. highlighted regional heterogeneity: while northern Xinjiang populations show substantial Western Eurasian contributions, southern populations align more closely with the Ancient North Eurasian lineage (Zhang, et al. 2021). Our extensive population genetic analyses confirmed Western Eurasian admixture in both ancient and present-day northwestern Chinese groups, with Northwest ethnic minorities having more Western Eurasian ancestry than Northwest Han Chinese. The Neolithic to Bronze Age populations in the Gonghe Plain and along the YRB formed primarily through admixture with local lineages, supplemented by gene flow from the Yangtze River or southern Siberia, without substantial input from Iranian farmers or steppe herders (Wang, et al. 2023). These findings suggest that the westward transmission of barley agriculture and Bronze Age cultural traits likely occurred through limited gene flow rather than large-scale population movement. Nonetheless, trace amounts of Western Eurasian ancestry have been detected in populations from the Hexi Corridor and its vicinity, potentially introduced by Sarmatians or their descendants during the Iron Age or later periods, as supported by *f*-statistical analysis. However, the limited availability of Iron Age and historical genomic data from the Hexi Corridor constrains our ability to resolve the timing and extent of this gene flow (Xiong, et al. 2024). Future studies with denser temporal sampling are essential for refining our understanding of the genetic influence of Western Eurasia in northwestern China. Deep demographic modeling based on the uniparental markers would also provide a window to explore new genomic insights of eastern TP populations (Wang, et al. 2024; Luo, et al. 2025). This refined understanding underscores the complex genetic and cultural interplay that shaped the population dynamics of this region.

## Conclusions

The TP and its surrounding corridors, among the last regions in Eurasia colonized by anatomically and behaviorally modern humans, present ongoing questions about the origins and migration routes of ancient and present-day highland populations. By generating WGS data for 293 individuals and compiling genome-wide SNP data from 799 Sino-Tibetan speakers, we provide new insights into the genetic structure of these populations. Our findings revealed pronounced genetic differentiation among Tibeto-Burman groups within the TYC and between the southwestern and northwestern subgroups. The influence of YRB millet farmers emerged as a major driver of genetic diversity across the highland TP, the TYC, and adjacent lowland regions. Multiple ancestral sources, including lineages from the YRB, South China, and western Eurasia, contributed to the complex genetic landscape of populations in the Tibetan-Yi and Hexi corridors. Demographic modeling indicated that Tibetan populations in the TYC shared a more common ancestor with Tibetans from the TP and lowland Sichuan than with neighboring Mosuo and Yi groups did. Notably, Tibetans from the TP and lowland Sichuan likely diverged ∼1.2 kya, indicating a relatively recent population split. This highlights the intricate demographic history of the TYC region. In the Hexi Corridor, modern populations derived most of their ancestry from ancient millet farming groups, with limited Western Eurasian input, reflecting early cultural diffusion and transcontinental exchange. Collectively, these findings refine our understanding of the demographic past in the Tibetan-Yi and Hexi corridors and underscore the multifaceted processes of human migration and interaction that have shaped the genetic and cultural landscape of this region.

## Materials and Methods

### Background of the Tibetan-Yi and Hexi Corridors and Sample Collection

The TYC, which is located primarily in the alpine valley regions of Sichuan, Yunnan, Chongqing, and Tibet, traverses the eastern edge of the TP. This corridor serves as a major route for human migration and settlement, significantly contributing to the genetic heritage of Sino-Tibetan-speaking populations in the area. Its geography is marked by a series of north-south mountains and rivers, defining its rugged terrain. Sichuan and Chongqing, which are landlocked regions in Southwest China, include the eastern Sichuan Basin and mountain ranges on the easternmost edge of the TP. These regions share borders with the Tibetan Autonomous Region, Qinghai, Gansu, Shaanxi, Guizhou, and Yunnan and are noted for their significant minority populations, including the Tibetan, Yi, Qiang, and Nakhi communities, which are primarily concentrated along the TYC. Yunnan, another landlocked province in Southwest China, borders Guizhou, Sichuan, Guangxi, the Tibetan Autonomous Region, and several Southeast Asian countries. The province's topography ranges from low altitudes in the southeast to high altitudes in the northwest, with the TYC situated in northwestern Yunnan. Yunnan is distinguished by its diversity, hosting the highest number of ethnic groups among all Chinese provinces and autonomous regions. The ethnic groups in its mountainous northwestern regions include Tibetans, Nakhi, Lisu, Pumi, and Drung. This geographical and demographic background is essential for understanding the complexities that influence genetic studies conducted in these corridors.

The Hexi Corridor, which is located in western Gansu Province, serves as a crucial historical gateway linking inland China with the Western Region. As a critical segment of the northern Silk Road, this corridor has been instrumental in facilitating human migration and cultural exchanges between East and West Eurasia (Yao, et al. 2021; Xiong, et al. 2022; He, et al. 2025). Strategically positioned along the narrowest part of Gansu Province, often referred to as the “neck” of the province, the Hexi Corridor has historically functioned as a vital strategic outpost and transportation hub. Gansu, a landlocked region in Northwest China, is bordered by Xinjiang and Qinghai to the west, Ningxia and Inner Mongolia to the north, Shaanxi to the East, and Sichuan to the south. The province lies between the Loess Plateau and the TP, adding to its geographical significance. Although Han Chinese constitute approximately 92% of its population, Gansu is also home to a diverse array of ethnic minorities, including Hui, Tibetan, Dongxiang, Tu, Yugur, Mongolian, Salar, Kazakh, Manchu, and Bonan, each contributing to the rich cultural tapestry of the region. This backdrop underscores the Hexi Corridor's role not only as a critical conduit for historical movements and interactions but also as a living mosaic of diverse cultures and traditions, reflecting the deep historical connections that have shaped this unique geographical and cultural landscape.

This study was approved by the Ethics Committees of Xiamen University and Sichuan University and complied with the ethical guidelines of the Helsinki Declaration of 2013 (World Medical Association 2013). Peripheral blood or saliva samples were collected from 799 Sino-Tibetan speakers for high-density SNP genotyping, including 458 Sinitic speakers from 36 populations and 341 Tibeto-Burman speakers from 24 populations. The demographic breakdown of the participants included 247 Sinitic people from 18 populations and 212 Tibeto-Burman people from 9 populations in Sichuan; 86 Sinitic people from 7 populations and 103 Tibeto-Burman people from 11 populations in Yunnan; 125 Sinitic people from 11 populations and 10 Tibeto-Burman people from Gansu; and 16 Tibeto-Burman people from 3 populations in surrounding provinces (Fig. 1a; Table S1). Forty-six representative individuals from four Tibeto-Burman-speaking populations—Muli Tibetan, Dujiangyan Tibetan, Xichang Yi, and Yanyuan Mosuo in Sichuan Province—were selected from the initial set of 799 array-sequenced samples for WGS (Fig. 1a). Additionally, 247 Tibeto-Burman-speaking individuals from the Huaxi Biobank were chosen for high-depth sequencing, representing participants from Sichuan Tibetan (Ganzi: 19; Aba: 5), Xizang Tibetan (Lhasa: 50), Gansu Tibetan (Lanzhou: 57), Qinghai Tibetan (Xining: 9; Yushu: 4), Guizhou Yi (Guiyang: 9), Sichuan Yi (Leshan: 37; Liangshan: 14), Yunnan Yi (Baoshan: 8; Chuxiong: 6; Honghe: 4; Kunming: 6; Pu’er: 5; Zhaotong: 8), Yunnan Hani (Pu'er: 5), and Sichuan Qiang (Aba: 1). Written informed consent was obtained from all participants, who were either indigenous to the sampling locations or had resided there for at least three generations. The sample selection was based on self-identified ethnicities and birthplaces, ensuring a diverse and representative sample for genetic analysis.

### Sequencing, Genotyping, and Data Analysis

#### DNA Extraction, Genotyping, and Quality Control

Genomic DNA was extracted from all the samples using the QIAamp DNA Mini Kit (QIAGEN, Germany) following the manufacturer's protocol. DNA concentrations were measured using the Qubit dsDNA HS Assay Kit on a Qubit 3.0 fluorometer (Thermo Fisher Scientific). Approximately 500,000 genome-wide SNPs were genotyped in 799 Tibeto-Burman-speaking individuals using the Affymetrix WeGene Array, which is designed explicitly for genetic studies of East Asian populations. We used PLINK v.1.9 to conduct quality controls on the array-based raw genomic data (Chang, et al. 2015). Samples were included if they met the following criteria: a genotyping success rate exceeding 99%, a minor AF above 0.01, and a Hardy-Weinberg equilibrium *P*-value greater than 0.001. Relatedness among individual pairs was assessed with both PLINK and KING, allowing precise estimation of familial relatedness within our dataset (Manichaikul, et al. 2010). Additionally, outliers were identified and removed using Genome-Wide Complex Trait Analysis (GCTA) v.1.93.2. Following these stringent quality control steps, 760 individuals were retained for subsequent analyses. This rigorous filtering process ensures the reliability and validity of the genetic data, providing a robust foundation for deriving deeper genetic insights from the study population.

#### Library Preparation and Sequencing

WGS was performed on 293 Tibeto-Burman-speaking individuals using the MGISEQ-2000 platform. To ensure result integrity, a blank control (water) was included at each stage of the wet laboratory procedures, from DNA extraction to sequencing. Sequencing libraries were prepared from double-stranded DNA following established protocols and indexed through dual parallel PCRs with Q5 High-Fidelity DNA Polymerase (New England Biolabs, United States). The PCR products from each library batch were pooled and purified via Agencourt AMPure XP beads (Beckman Coulter, Germany). After purification, the libraries were eluted in TET buffer and quantified via a Qubit 2.0 Fluorometer (Thermo Fisher, United States). Finally, the enriched libraries were sequenced on the MGISEQ-2000 platform at Annoroad Gene Technology (Beijing, China) using a 150 bp paired-end sequencing strategy.

#### Processing of Whole-Genome Sequencing Data

We sequenced 46 representative samples previously genotyped with the Affymetrix array to a depth of 10× and all samples from the Huaxi Biobank to a depth of 50×. We aligned the read pairs to the human reference genome GRCh37 (human_g1k_v37) using BWA-MEM v.0.7.13 (Li 2013). To ensure data quality, PCR duplicates were removed using Dedup v.0.12.3 (Peltzer, et al. 2016). The aligned sequencing reads from various lanes were consolidated and organized using SAMtools v.1.5 (Li, et al. 2009), followed by base quality recalibration with BamUtil recab v.1.3. Joint genotype calling was conducted using the HaplotypeCaller module of the Genome Analysis Toolkit (GATK) v.4.1.4.1 (McKenna, et al. 2010; DePristo, et al. 2011). This multistep processing pipeline ensures that the sequencing data are optimally prepared for comprehensive genomic analyses, thereby increasing the reliability of the genetic findings.

#### Kinship Identification

The genetic sex of all the sampled individuals was determined by computing the coverage ratios of the X and Y chromosomes relative to the autosomes. The relatedness between pairs of whole-genome sequenced individuals was assessed by calculating the pairwise mismatch rate, which quantifies genome-wide allele mismatches at overlapping genetic loci. This method reliably assesses genetic relationships, enabling the identification of direct kinship ties, including twins and first- and second-degree relatives (Monroy Kuhn et al. 2018).

### Population Structure Analyses

#### Dataset Integration

To enhance our understanding of the genetic structure of the Tibetan-Yi and Hexi Corridor populations, we integrated newly generated genotyping data with two extensive genotype panels. The first panel, derived from the HO dataset, contains approximately 600,000 SNPs and is sourced from the Allen Ancient DNA Resource (Mallick, et al. 2024). The second panel, the 1240K dataset, includes all the SNPs in the HO dataset (Mallick, et al. 2024). Our core dataset, consisting of 760 array-genotyped and 293 whole-genome sequenced individuals, was combined with an East Asian panel featuring genotyping data for 2,298 individuals across 188 populations, all processed using the Affymetrix WeGene V1 Array (He, et al. 2021; Li, et al. 2024). To enable comprehensive population genetic analyses, we merged the integrated Affymetrix dataset with the HO dataset, creating expanded HO datasets (HO_Affy and HO_Affy_WGS). Similarly, merging the Affymetrix dataset with the 1240K dataset resulted in an expanded 1240K dataset (1240K_Affy). To investigate the fine-scale genetic structure of the study population within a broader spatiotemporal framework and at higher SNP density, we merged the newly generated WGS dataset with the HO dataset to create an additional expanded dataset (HO_WGS). Additionally, to reconstruct the demographic history of populations in the Tibetan-Yi and Hexi corridors under complex evolutionary scenarios, we integrated our WGS data with high-depth sequences from the Human Genome Diversity Project (HGDP), Oceanian genomic resources, and the pilot phase of the 10K Chinese People Genomic Diversity Project (Bergström et al. 2020; Choin, et al. 2021; He, et al. 2025). These integrations enhance the resolution of genetic diversity and historical population dynamics by leveraging both contemporary and ancient genomic data across diverse geographic and temporal scales.

#### PCA and ADMIXTURE Modeling

To investigate the genetic structure of the newly genotyped populations, we performed PCA using the smartpca package from EIGENSOFT (Patterson, et al. 2012). We projected ancient individuals onto contemporary populations, optimizing parameters to minimize outliers (numoutlieriter: 0) and ensuring accurate least-squares projection (lsqproject: YES). We also calculated pairwise *F*_ST_ values to quantify genetic differentiation between the studied groups and various Eurasian reference populations using PLINK v.1.9 (Chang, et al. 2015). Furthermore, we performed a model-based unsupervised clustering analysis using ADMIXTURE v.1.3.0 (Alexander, et al. 2009). SNPs exhibiting strong linkage disequilibrium were pruned using PLINK with the parameters −indep-pairwise 200 25 0.4 to ensure marker independence (Chang, et al. 2015). The ADMIXTURE analysis was conducted across a range of hypothetical ancestral contributions (*K* values from 2 to 20), with 10-fold cross-validation (−cv = 10) to evaluate model accuracy and stability based on the pruned dataset.

#### F-Statistics

To assess shared genetic drift and detect potential admixture signals within our targeted populations, we used two types of *f_3_*-statistics using the Qp3pop package from ADMIXTOOLS (Patterson, et al. 2012). These analyses, expressed as *f_3_* (Source1, Source2; Target/Mbuti), are instrumental in evaluating the genetic relationships and admixture events among the populations under study. We further explored asymmetric shared ancestry with the qpDstat package, utilizing the *f_4_* model (*f_4_*Mode: YES) to dissect genetic interactions and lineage distinctions (Patterson, et al. 2012).

#### Phylogenetic Relationship Reconstruction and TreeMix Analysis

To elucidate the phylogenetic relationships among the studied populations, we constructed neighbor-joining trees based on *F*_ST_ values and the transformed *f_3_* genetic matrix using MEGA 7.0 (Kumar, et al. 2016). Additionally, we used TreeMix (Pickrell and Pritchard 2012) to construct a maximum likelihood (ML) tree, which allowed us to infer the patterns of population splits and mixtures between target populations and reference groups. This approach facilitated the visualization of evolutionary histories inferred from genetic data, offering further insights into population divergence and gene flow.

#### QpWave and QpAdm Analyses

To infer the number of admixture events, test how a single target population relates to reference populations, and estimate admixture weights, we performed qpWave/qpAdm analysis using ADMIXTOOLS (Patterson, et al. 2012). Our analysis included a diverse set of outgroups from Eastern and Western Eurasian as well as non-Eurasian lineages, incorporating both ancient individuals and present-day proxies such as Mbuti, GanjDareh_N, Villabruna_HG, Mixe, Papuan, Onge, and Atayal (Jeong, et al. 2019; Wang, et al. 2021a). This comprehensive approach refined our admixture models, deepening our understanding of ancestry sources and their proportions within the populations studied. By integrating a broad spectrum of genetic backgrounds, we precisely delineated the contributions of diverse ancestral lineages to the genetic makeup of our target groups.

#### ALDER Analysis

We used ALDER to infer the timing and nature of admixture events within the study populations (Loh, et al. 2013). To estimate admixture dates for genetically diverse groups from the Tibetan-Yi and Hexi corridors, we conducted serial modeling with a broad range of eastern and western Eurasian source populations. These included southern East Asian sources (Ami, Atayal, BoY, Dao, Dong_Guizhou, Hmong), northern East Asian sources (Han_Henan, Han_Shandong, Nanai, Nganasan, Tibetan_Lhasa, Ulchi), and western Eurasian sources (Spanish, Turkish, Armenian, Sardinian, French, Greek, Tajik_Mountain, Basque). By comparing recipient populations with these predefined ancestral sources, we estimated admixture timings and focused on characterizing single-pulse admixture events. This approach simplified complex historical intermixtures and clarified the genetic chronology of population formation.

#### Haplotype-Based Fine-Scale Population Structure Dissection

We phased autosomal SNP data using ShapeIT v.2.0, adhering to the recommended human genetic maps (Delaneau, et al. 2012). Refined IBD (Browning and Browning 2013) was applied to reconstruct shared identity by descent segments, both in terms of number and length, at the individual and population levels. We used fineSTRUCTURE v4 (Lawson, et al. 2012) to further explore the fine-scale population structure. This tool enables us to assess genetic homogeneity or heterogeneity across geographically classified populations and to reconstruct phylogenetic relationships based on shared coancestry matrices (Lawson, et al. 2012). Furthermore, CHROMOPAINTERv2 was applied to paint recipient populations’ chromosomes as haplotype chunks, estimating the number of ancestry chunks inherited from donor populations. Finally, GLOBETROTTER was used to date admixture events, considering various plausible surrogates or donor populations (Hellenthal, et al. 2014).

#### Effective Population Size and Divergence Time Estimation

We inferred population size histories using SMC++ (Terhorst, et al. 2017) based on biallelic SNP genotypes from all individuals within each population. We ran SMC++ with a mutation rate of 1.25 × 10^−8^ per base pair per generation and assumed a generation time of 29 years. To reconstruct population divergence histories, we performed a series of MSMC2 analyses (Schiffels and Wang 2020). We estimated split times between newly sequenced and linguistically distinct Chinese reference populations by selecting four individuals per population from the following groups: Tibeto-Burman-speaking Anha Yi, Amdo Tibetan, and Baima Tibetan (Sichuan); Zhuoni Tibetan (Gansu); Sinitic-speaking Sichuan Hui, and northern and southern Han Chinese; Altaic-speaking Daur and Manchu (Inner Mongolia); Austroasiatic-speaking Blang and Wa (Yunnan); Austronesian-speaking Gaoshan (Fujian and Taiwan); Hmong-Mien-speaking Sichuan Miao and Fujian She; and Tai-Kadai-speaking Guizhou Bouyei and Hainan Li. For populations with fewer than four individuals, we included all available samples. We adopted the same mutation rate and generation time parameters used in the SMC++ analysis to estimate the divergence times.

#### Complex Demographic History Inference

We investigated the demographic history of TYC populations and Han Chinese groups using coalescent simulations implemented in fastsimcoal v2.7 (Excoffier, et al. 2013), which estimates demographic parameters through a composite likelihood approach. We filtered SNPs based on the following criteria: (i) missing in at least one sample; (ii) deviating from Hardy-Weinberg equilibrium (*P* < 10⁻^4^) in at least one population; (iii) located within CpG islands; (iv) located in coding regions; and (v) lacking ancestral state information. We then generated multidimensional site frequency spectra (SFS) using the ProcessVCF_bootstrap.sh script (https://cmpg.unibe.ch/software/fastsimcoal2/additionalScripts.html). Based on previous findings, we designed six demographic models to estimate population splits and divergence times. First, we constructed three models using 4D-SFS to explore the relationships among Sino-Tibetan groups—Northern Han, Southern Han, Muli Tibetan in the TYC, and Lhasa Tibetan in the TP. We compared models with differing phylogenetic structures: (i) geographically distinct Tibetan groups sharing a common ancestry; (ii) TYC Tibetans sharing ancestry with Han Chinese; and (iii) TYC Tibetans sharing ancestry with Han Chinese plus gene flow from highland Tibetans. After identifying the best-fitting base model, we developed three additional models using 6D-SFS to explore the relationships between the TYC groups (Tibetan_Dujiangyan, Tibetan_Muli, Yi_Xichang, and Mosuo_Yanyuan) and other Tibeto-Burman groups (Northern Han, Southern Han, Tibetan_Lhasa). We tested the following scenarios: (i) Mosuo and Yi share ancestry with Tibetan groups; (ii) Mosuo and Yi share ancestry with Han Chinese; and (iii) Mosuo and Yi share ancestry with Muli Tibetan from the TYC. Each model underwent 600,000 simulations with 65 expectation conditional maximization optimization cycles and 100 replicate runs initialized from different random seeds. We computed and optimized the likelihood using both polymorphic and monomorphic sites during the first 25 cycles (“-l 25”) (Choin, et al. 2021). To avoid overfitting, we retained only SFS entries with counts >5 for parameter estimation (“-C 5”). To determine the ML estimate for each model, we selected the best-performing run among the 100 replicates. We then reestimate the likelihood for each best run using 100 expected SFS values derived from 600,000 simulations. Specifically, we calculated the log_10_(likelihood) using 10⁷ simulations and designated the run with the highest likelihood as the ML estimate. We rescaled the demographic parameters to correct for discrepancies in SNP counts between the observed and expected SFS using a rescaling factor. We assumed a generation time of 29 years and a mutation rate of 1.25 × 10⁻^8^ per generation per site for all time estimates. For model selection, we compared models based on differences in the expected log_10_(likelihood) of the observed SFS (initial likelihood) rather than using the Akaike information criterion. A model was considered the best fit if (i) its initial expected log_10_(likelihood) exceeded that of alternatives and (ii) the mean log_10_(likelihood) difference between this model and alternatives exceeded 50 across 100 reestimations. We calculated CIs using a nonparametric block bootstrap approach. We generated 100 bootstrapped datasets by randomly resampling 1 Mb genomic blocks with replacement, maintaining the same total block count as in the original data. We reestimated parameters using ML values across 20 replicate runs per bootstrap. We defined 95% CIs as the 2.5th and 97.5th percentiles of the resulting parameter distributions.

## Supplementary Material

msaf258_Supplementary_Data

## Data Availability

All the data required to evaluate the conclusions of this paper are provided within the main text and/or supplementary materials. The alignment files and microarray-based genotyping data are available at the Genome Warehouse in the National Genomics Data Center, Beijing Institute of Genomics (China National Center for Bioinformation), Chinese Academy of Sciences, under accession numbers GVM000852, OMIX011779, OMIX011781-01, and OMIX011781-02. These files can be accessed publicly at https://ngdc.cncb.ac.cn/gvm/. Zenodo, with the accession number 15410955, also provided data access. The National Health Commission of the People's Republic of China authorized the use of genetic data for this study (no. 2025BAT00384). Further information can be obtained from the corresponding authors. Haploid genotype data for ancient individuals in this study, which are based on the 1240K panel, are available in EIGENSTRAT format at the following link: https://dataverse.harvard.edu/dataset.xhtml?persistentId = doi:10.7910/DVN/FFIDCW.

## References

[msaf258-B1] Alexander DH, Novembre J, Lange K. Fast model-based estimation of ancestry in unrelated individuals. Genome Res. 2009:19:1655–1664. 10.1101/gr.094052.109. 19648217 PMC2752134

[msaf258-B2] Bai F et al Ancient genomes revealed the complex human interactions of the ancient western Tibetans. Curr Biol. 2024:34:2594–2605.e7. 10.1016/j.cub.2024.04.068. 38781957

[msaf258-B3] Bergström A et al Insights into human genetic variation and population history from 929 diverse genomes. Science. 2020:367:1339. 10.1126/science.aay5012. PMC7115999 32193295

[msaf258-B4] Browning BL, Browning SR. Improving the accuracy and efficiency of identity-by-descent detection in population data. Genetics. 2013:194:459–471. 10.1534/genetics.113.150029. 23535385 PMC3664855

[msaf258-B5] Chang CC et al Second-generation PLINK: rising to the challenge of larger and richer datasets. Gigascience. 2015:4:7. 10.1186/s13742-015-0047-8. 25722852 PMC4342193

[msaf258-B6] Chen F et al A late middle Pleistocene Denisovan mandible from the Tibetan Plateau. Nature. 2019:569:409–412. 10.1038/s41586-019-1139-x. 31043746

[msaf258-B7] Chen FH et al Agriculture facilitated permanent human occupation of the Tibetan plateau after 3600 B.P. Science. 2015:347:248–250. 10.1126/science.1259172. 25593179

[msaf258-B8] Choin J et al Genomic insights into population history and biological adaptation in oceania. Nature. 2021:592:583–589. 10.1038/s41586-021-03236-5. 33854233

[msaf258-B9] Cong P-K et al Genomic analyses of 10,376 individuals in the Westlake BioBank for Chinese (WBBC) pilot project. Nat Commun. 2022:13:2939. 10.1038/s41467-022-30526-x. 35618720 PMC9135724

[msaf258-B10] Delaneau O, Marchini J, Zagury J-F. A linear complexity phasing method for thousands of genomes. Nat Methods. 2012:9:179–181. 10.1038/nmeth.1785. 22138821

[msaf258-B11] DePristo MA et al A framework for variation discovery and genotyping using next-generation DNA sequencing data. Nat Genet. 2011:43:491–498. 10.1038/ng.806. 21478889 PMC3083463

[msaf258-B12] Ding M et al Ancient mitogenomes show plateau populations from last 5200 years partially contributed to present-day Tibetans. Proc Biol Sci. 2020:287:20192968. 10.1098/rspb.2019.2968. 32183622 PMC7126037

[msaf258-B13] Excoffier L, Dupanloup I, Huerta-Sánchez E, Sousa VC, Foll M. Robust demographic inference from genomic and SNP data. PLoS Genet. 2013:9:e1003905. 10.1371/journal.pgen.1003905. 24204310 PMC3812088

[msaf258-B14] Feng Q et al Genetic history of Xinjiang's Uyghurs suggests Bronze Age multiple-way contacts in Eurasia. Mol Biol Evol. 2017:34:2572–2582. 10.1093/molbev/msx177. 28595347

[msaf258-B15] He G et al Fine-scale genetic structure of Tujia and central Han Chinese revealing massive genetic admixture under language borrowing. J Syst Evol. 2021:59:1–20. 10.1111/jse.12670.

[msaf258-B16] He G et al Pilot work of the 10 K Chinese People Genomic Diversity Project along the Silk Road suggests a complex east-west admixture landscape and biological adaptations. Sci China Life Sci. 2025:68:914–933. 10.1007/s11427-024-2748-4. 39862346

[msaf258-B17] He K, Lu H, Zhang J, Wang C, Huan X. Prehistoric evolution of the dualistic structure mixed rice and millet farming in China. Holocene. 2017:27:1885–1898. 10.1177/0959683617708455.

[msaf258-B18] Hellenthal G et al A genetic atlas of human admixture history. Science. 2014:343:747–751. 10.1126/science.1243518. 24531965 PMC4209567

[msaf258-B21] Jeong C et al A dynamic 6,000-year genetic history of Eurasia's eastern steppe. Cell. 2020:183:890–904.e29. 10.1016/j.cell.2020.10.015. 33157037 PMC7664836

[msaf258-B19] Jeong C et al Long-term genetic stability and a high-altitude East Asian origin for the peoples of the high valleys of the Himalayan arc. Proc Natl Acad Sci U S A. 2016:113:7485–7490. 10.1073/pnas.1520844113. 27325755 PMC4941446

[msaf258-B20] Jeong C et al The genetic history of admixture across inner Eurasia. Nat Ecol Evol. 2019:3:966–976. 10.1038/s41559-019-0878-2. 31036896 PMC6542712

[msaf258-B22] Kumar S, Stecher G, Tamura K. MEGA7: molecular evolutionary genetics analysis version 7.0 for bigger datasets. Mol Biol Evol. 2016:33:1870–1874. 10.1093/molbev/msw054. 27004904 PMC8210823

[msaf258-B23] Kumar V et al Bronze and Iron Age population movements underlie Xinjiang population history. Science. 2022:376:62–69. 10.1126/science.abk1534. 35357918

[msaf258-B24] Lawson DJ, Hellenthal G, Myers S, Falush D. Inference of population structure using dense haplotype data. PLoS Genet. 2012:8:e1002453. 10.1371/journal.pgen.1002453. 22291602 PMC3266881

[msaf258-B25] Lazaridis I et al Genomic insights into the origin of farming in the ancient Near East. Nature. 2016:536:419–424. 10.1038/nature19310. 27459054 PMC5003663

[msaf258-B27] Li H et al The sequence alignment/map format and SAMtools. Bioinformatics. 2009:25:2078–2079. 10.1093/bioinformatics/btp352. 19505943 PMC2723002

[msaf258-B26] Li H . Aligning sequence reads, clone sequences and assembly contigs with BWA-MEM. arXiv 1303.3997. 10.48550/arXiv.1303.3997, 16 March 2013, preprint: not peer reviewed.

[msaf258-B28] Li X et al Evolutionary history and biological adaptation of Han Chinese people on the Mongolian Plateau. hLife. 2024:2:296–313. 10.1016/j.hlife.2024.04.005.

[msaf258-B29] Liu C-C et al Ancient genomes from the Himalayas illuminate the genetic history of Tibetans and their Tibeto-Burman speaking neighbors. Nat Commun. 2022:13:1203. 10.1038/s41467-022-28827-2. 35260549 PMC8904508

[msaf258-B30] Liu J et al East Asian gene flow bridged by northern coastal populations over past 6000 years. Nat Commun. 2025:16:1322. 10.1038/s41467-025-56555-w. 39900598 PMC11791043

[msaf258-B31] Loh P-R et al Inferring admixture histories of human populations using linkage disequilibrium. Genetics. 2013:193:1233–1254. 10.1534/genetics.112.147330. 23410830 PMC3606100

[msaf258-B32] Luo L et al Sequencing and characterizing human mitochondrial genomes in the biobank-based genomic research paradigm. Sci China Life Sci. 2025:68:1610–1625. 10.1007/s11427-024-2736-7. 39843848

[msaf258-B33] Ma T, Rolett BV, Zheng Z, Zong Y. Holocene coastal evolution preceded the expansion of paddy field rice farming. Proc Natl Acad Sci U S A. 2020:117:24138–24143. 10.1073/pnas.1919217117. 32929013 PMC7533829

[msaf258-B34] Mallick S et al The Allen Ancient DNA Resource (AADR) a curated compendium of ancient human genomes. Sci Data. 2024:11:182. 10.1038/s41597-024-03031-7. 38341426 PMC10858950

[msaf258-B35] Manichaikul A et al Robust relationship inference in genome-wide association studies. Bioinformatics. 2010:26:2867–2873. 10.1093/bioinformatics/btq559. 20926424 PMC3025716

[msaf258-B36] McKenna A et al The genome analysis toolkit: a MapReduce framework for analyzing next-generation DNA sequencing data. Genome Res. 2010:20:1297–1303. 10.1101/gr.107524.110. 20644199 PMC2928508

[msaf258-B37] Meyer MC et al Permanent human occupation of the central Tibetan Plateau in the early Holocene. Science. 2017:355:64–67. 10.1126/science.aag0357. 28059763

[msaf258-B38] Monroy Kuhn JM, Jakobsson M, Günther T. Estimating genetic kin relationships in prehistoric populations. PLoS One. 2018:13:e0195491. 10.1371/journal.pone.0195491. 29684051 PMC5912749

[msaf258-B40] Ning C et al Ancient genomes from northern China suggest links between subsistence changes and human migration. Nat Commun. 2020:11:2700. 10.1038/s41467-020-16557-2. 32483115 PMC7264253

[msaf258-B39] Ning C et al Ancient genomes reveal Yamnaya-related ancestry and a potential source of Indo-European speakers in Iron Age Tianshan. Curr Biol. 2019:29:2526–2532.e4. 10.1016/j.cub.2019.06.044. 31353181

[msaf258-B41] Pan Y et al Genomic diversity and post-admixture adaptation in the Uyghurs. Natl Sci Rev. 2022:9:nwab124. 10.1093/nsr/nwab124. 35350227 PMC8953455

[msaf258-B42] Patterson N et al Ancient admixture in human history. Genetics. 2012:192:1065–1093. 10.1534/genetics.112.145037. 22960212 PMC3522152

[msaf258-B43] Peltzer A et al EAGER: efficient ancient genome reconstruction. Genome Biol. 2016:17:60. 10.1186/s13059-016-0918-z. 27036623 PMC4815194

[msaf258-B44] Pickrell JK, Pritchard JK. Inference of population splits and mixtures from genome-wide allele frequency data. PLoS Genet. 2012:8:e1002967. 10.1371/journal.pgen.1002967. 23166502 PMC3499260

[msaf258-B45] Sagart L et al Dated language phylogenies shed light on the ancestry of Sino-Tibetan. Proc Natl Acad Sci U S A. 2019:116:10317–10322. 10.1073/pnas.1817972116. 31061123 PMC6534992

[msaf258-B46] Schiffels S, Wang K. MSMC and MSMC2: the multiple sequentially Markovian coalescent. Methods Mol Biol. 2020:2090:147–166. 10.1007/978-1-0716-0199-0_7. 31975167

[msaf258-B47] Shi H et al Y chromosome evidence of earliest modern human settlement in East Asia and multiple origins of Tibetan and Japanese populations. BMC Biol. 2008:6:45. 10.1186/1741-7007-6-45. 18959782 PMC2605740

[msaf258-B48] Terhorst J, Kamm JA, Song YS. Robust and scalable inference of population history from hundreds of unphased whole genomes. Nat Genet. 2017:49:303–309. 10.1038/ng.3748. 28024154 PMC5470542

[msaf258-B49] The 1000 Genomes Project Consortium . A global reference for human genetic variation. Nature. 2015:526:68–74. 10.1038/nature15393. 26432245 PMC4750478

[msaf258-B50] Wang C-C et al Genomic insights into the formation of human populations in east Asia. Nature. 2021a:591:413–419. 10.1038/s41586-021-03336-2. 33618348 PMC7993749

[msaf258-B51] Wang H et al Human genetic history on the Tibetan Plateau in the past 5100 years. Sci Adv. 2023:9:eadd5582. 10.1126/sciadv.add5582. 36930720 PMC10022901

[msaf258-B52] Wang M et al Multiple human population movements and cultural dispersal events shaped the landscape of Chinese paternal heritage. Mol Biol Evol. 2024:41:msae122. 10.1093/molbev/msae122. 38885310 PMC11232699

[msaf258-B53] Wang T et al Prehistoric genomes from Yunnan reveal ancestry related to Tibetans and Austroasiatic speakers. Science. 2025:388:eadq9792. 10.1126/science.adq9792. 40440384

[msaf258-B54] Wang W et al Ancient Xinjiang mitogenomes reveal intense admixture with high genetic diversity. Sci Adv. 2021b:7:eabd6690. 10.1126/sciadv.abd6690. 33789892 PMC8011967

[msaf258-B55] World Medical Association . World Medical Association Declaration of Helsinki: ethical principles for medical research involving human subjects. JAMA. 2013:310:2191–2194. 10.1001/jama.2013.281053. 24141714

[msaf258-B56] Xiong J et al Sex-biased population admixture mediated subsistence strategy transition of Heishuiguo people in Han Dynasty Hexi Corridor. Front Genet. 2022:13:827277. 10.3389/fgene.2022.827277. 35356424 PMC8960071

[msaf258-B57] Xiong J et al Inferring the demographic history of Hexi Corridor over the past two millennia from ancient genomes. Sci Bull (Beijing). 2024:69:606–611. 10.1016/j.scib.2023.12.031. 38184385

[msaf258-B58] Yang MA et al Ancient DNA indicates human population shifts and admixture in northern and southern China. Science. 2020:369:282–288. 10.1126/science.aba0909. 32409524

[msaf258-B59] Yao H et al New insights into the fine-scale history of western-eastern admixture of the northwestern Chinese population in the Hexi Corridor via genome-wide genetic legacy. Mol Genet Genomics. 2021:296:631–651. 10.1007/s00438-021-01767-0. 33650010

[msaf258-B60] Yue J-P et al Human adaptations during MIS 2: evidence from microblade industries of Northeast China. Palaeogeogr Palaeoclimatol Palaeoecol. 2021:567:110286. 10.1016/j.palaeo.2021.110286.

[msaf258-B61] Zhang D et al Denisovan DNA in Late Pleistocene sediments from Baishiya Karst Cave on the Tibetan Plateau. Science. 2020:370:584–587. 10.1126/science.abb6320. 33122381

[msaf258-B62] Zhang F et al The genomic origins of the Bronze Age Tarim basin mummies. Nature. 2021:599:256–261. 10.1038/s41586-021-04052-7. 34707286 PMC8580821

[msaf258-B63] Zhang M, Yan S, Pan W, Jin L. Phylogenetic evidence for Sino-Tibetan origin in northern China in the Late Neolithic. Nature. 2019:569:112–115. 10.1038/s41586-019-1153-z. 31019300

[msaf258-B64] Zhang XL et al The earliest human occupation of the high-altitude Tibetan Plateau 40 thousand to 30 thousand years ago. Science. 2018:362:1049–1051. 10.1126/science.aat8824. 30498126

[msaf258-B65] Zuo X et al Dating rice remains through phytolith carbon-14 study reveals domestication at the beginning of the Holocene. Proc Natl Acad Sci U S A. 2017:114:6486–6491. 10.1073/pnas.1704304114. 28559349 PMC5488950

