# Supplementary material for: Human Population Genetic History and Evolutionary Dynamics on the Eastern Tibetan Plateau
Source: Mol Biol Evol. 2025 Nov 17;42(11):msaf258. doi: 10.1093/molbev/msaf258 (PMC12620650; doi:10.1093/molbev/msaf258)
Supplement: msaf258_Supplementary_Data [file msaf258_supplementary_data.zip › Supplementary Figures.pdf]

Supplementary Figures for
Human population genetic history and evolutionary dynamics on the Eastern Tibetan Plateau

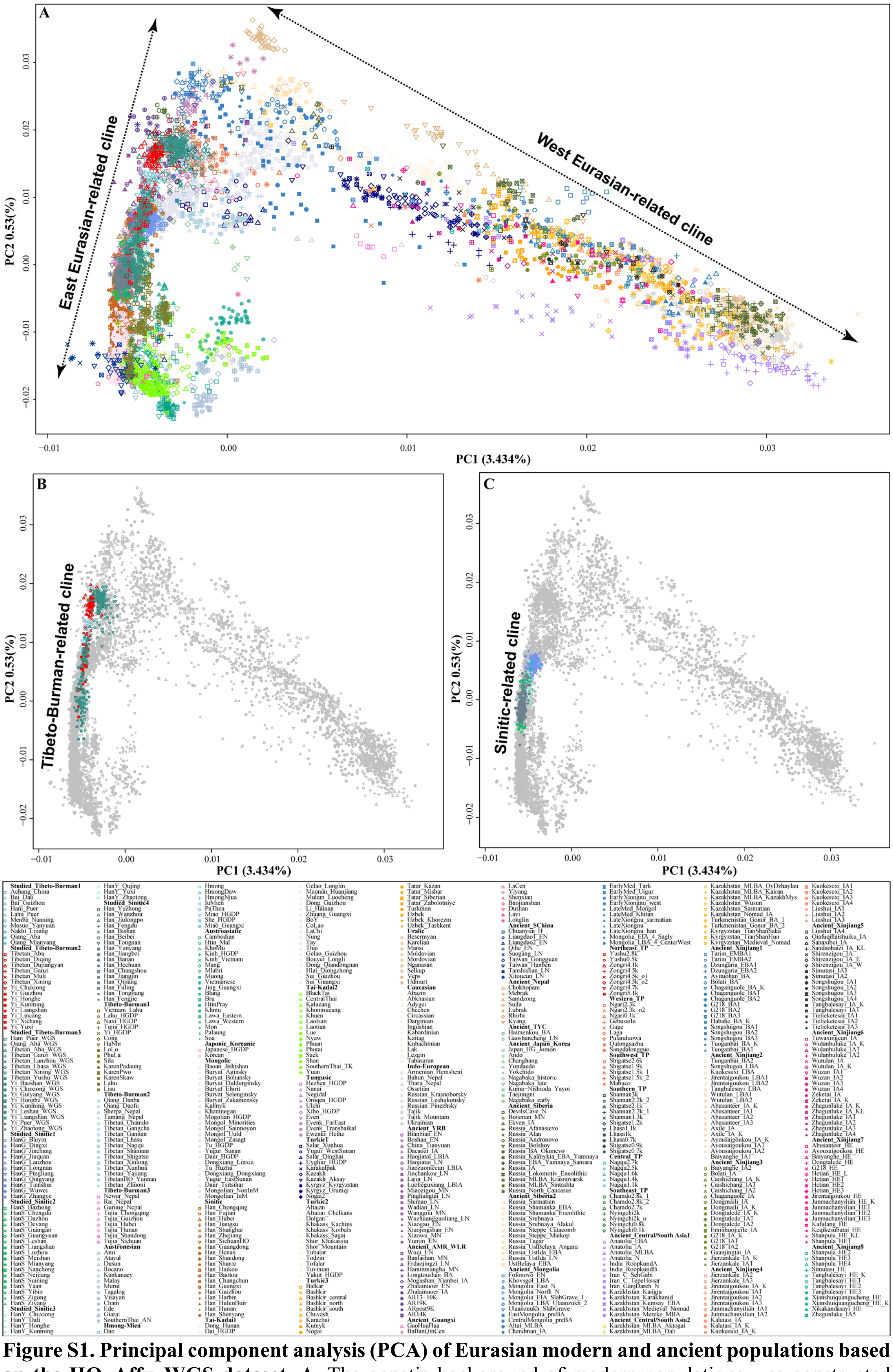

Figure S1. Principal component analysis (PCA) of Eurasian modern and ancient populations based on the HO_Affy_WGS dataset. A, The genetic background of modern populations was constructed based on the genetic variations of 5,779 individuals in 337 populations belonging to thirteen language

families: Sinitic, Tibeto-Burman, Austroasiatic, Austronesian, Tai-Kadai, Hmong-Mien, Japonic&Koreanic, Mongolic, Tungusic, Turkic, Indo-European, Uralic, and Caucasian. A total of 1,263 Paleolithic to historic ancient individuals from 318 populations were projected onto the genetic background based on the top components (PC1: 3.44% and 0.53%). **B**, Eurasian-level PCA with newly genotyped Tibeto-Burman groups being highlighted. **C**, Eurasian-level PCA with newly genotyped Sinitic-speaking populations being highlighted.

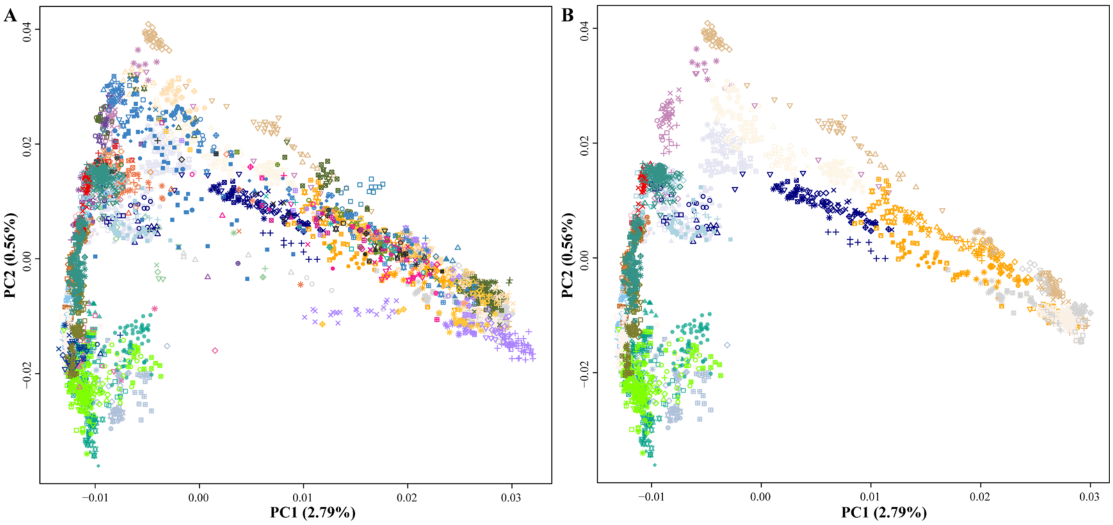

Figure S2. Genetic relationships between modern and ancient Eurasian populations inferred based on the HO_WGS dataset. A, Eurasian-scale PCA constructed based on 2,645 individuals from 231 modern populations covering thirteen language families and 1,289 ancient individuals from 330 populations in the merged HO_WGS dataset. B, The genetic background constructed based on the genetic variations of 231 modern Eurasian populations.

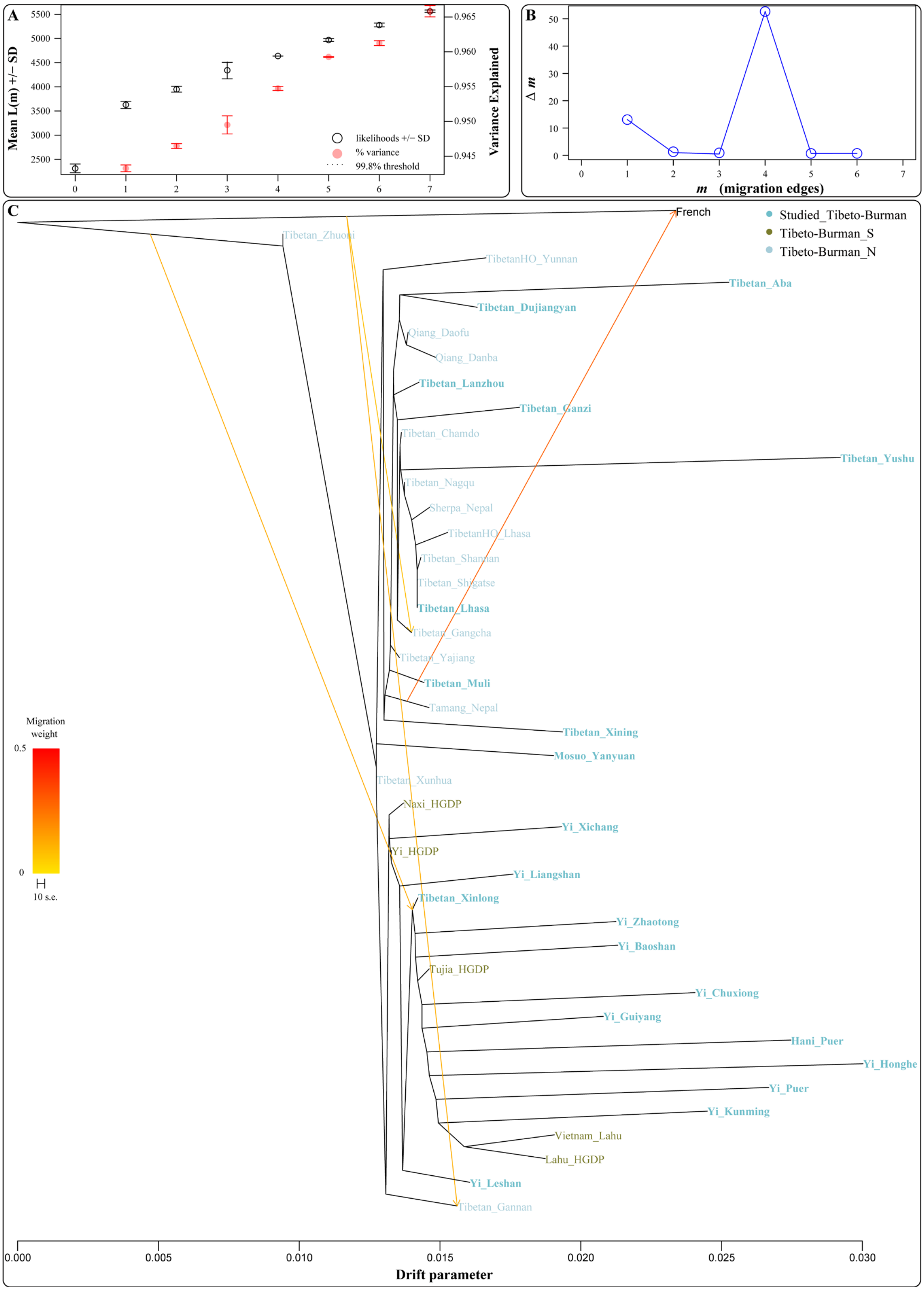

Figure S3. Population split and admixture patterns inferred from TreeMix analysis using the HO_WGS dataset. A, The mean and standard deviation (SD) of 10 iterations of the composite likelihood L(m) (left axis, black circles) and the proportion of explained variance (right axis, red "x"). **B**, The second-order rate of change (Δm) across m values. **C**, The maximum likelihood tree depicting four migration events among Tibeto-Burman-speaking populations included in the PCA shown in Figure 1c. The Aba Qiang (n = 1) was excluded, and the French population was used as the root. Population colors match those used in the PCA shown in Figure 1c.

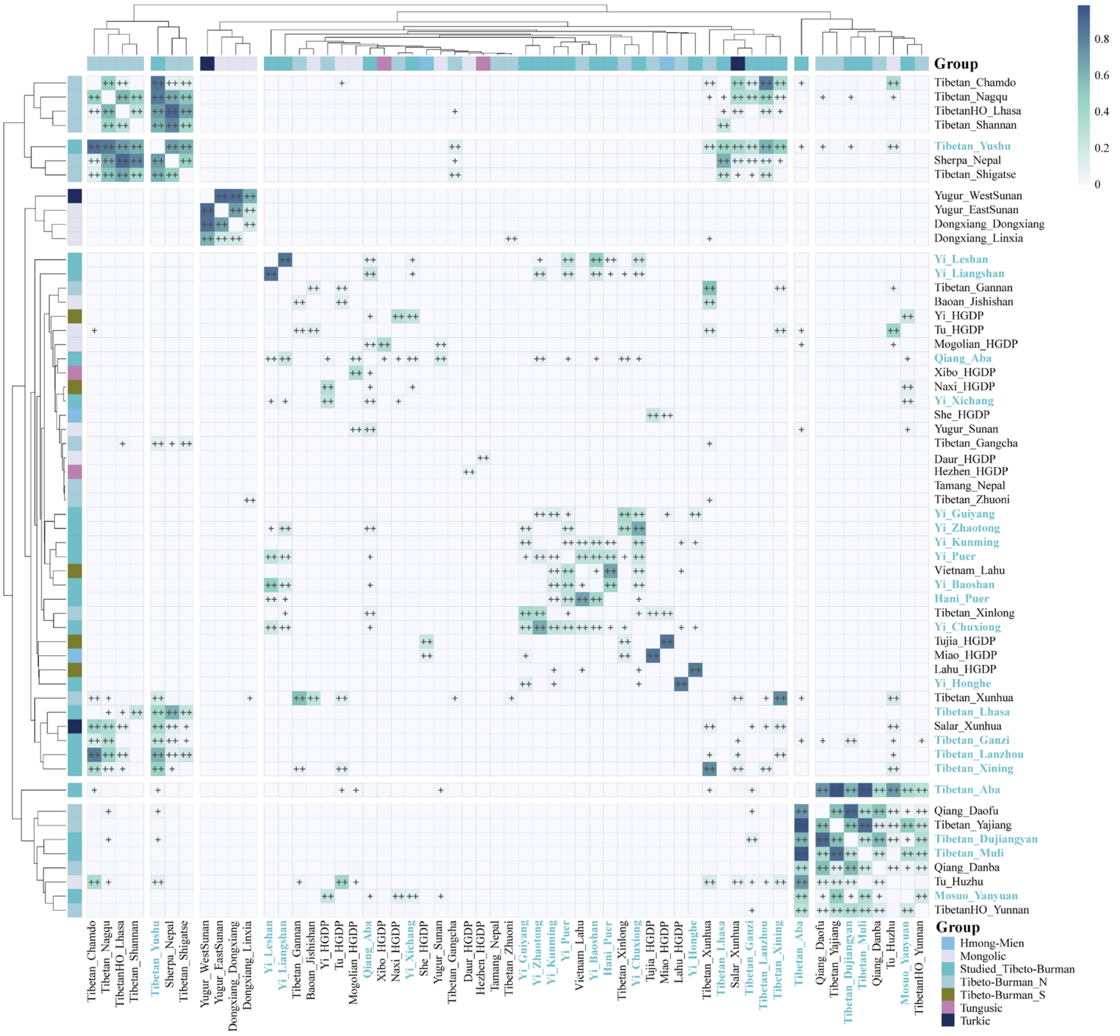

Figure S4. The qpWave analysis of modern East Eurasian populations based on the HO_WGS dataset. The cluster block colors for each population match those used in the PCA plot in Figure 1c.

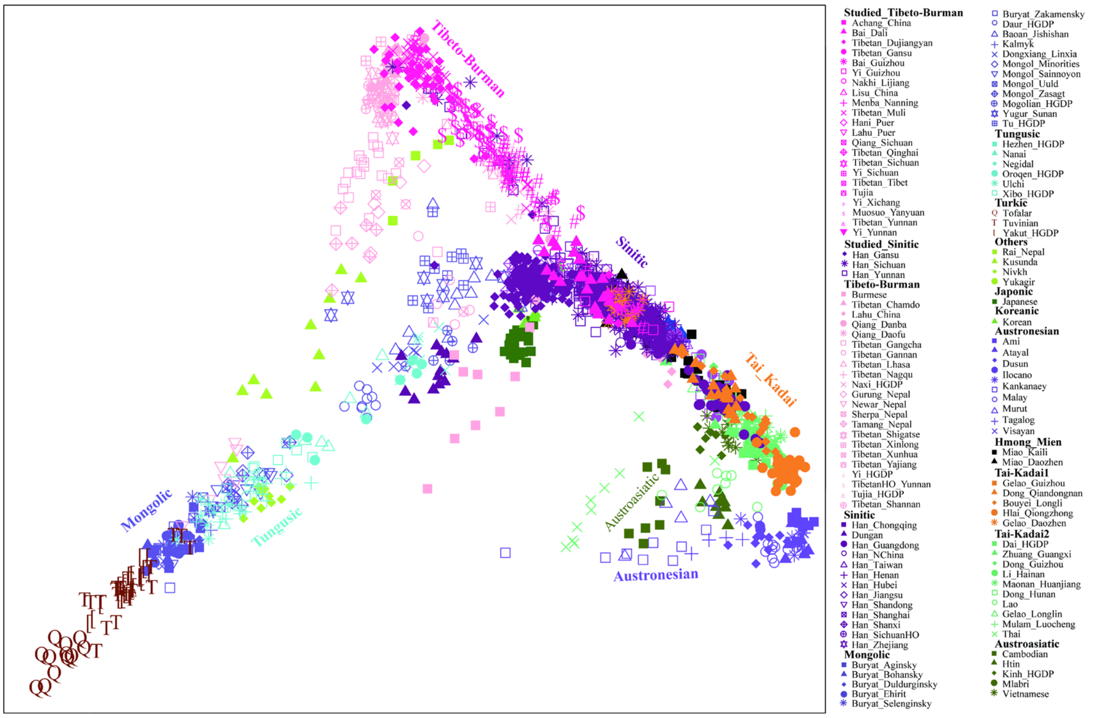

Figure S5. East Asian-scale PCA among 2,066 individuals based on the HO_Affy dataset.

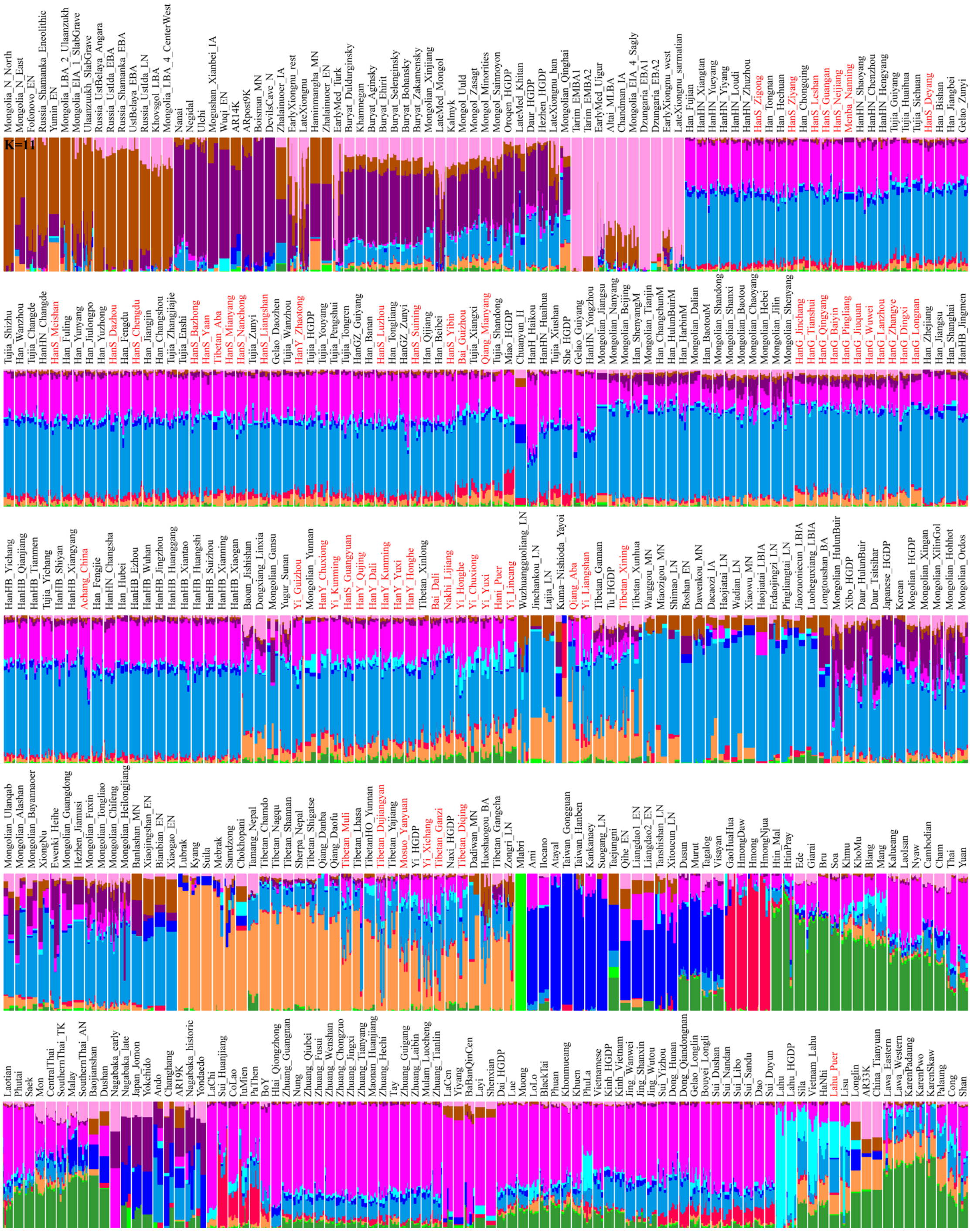

Figure S6. Model-based ADMIXTURE results for 4,167 ancient and modern individuals from 418 populations with eleven predefined ancestral sources based on the HO_Affy dataset. We visualized the best-fitting model with the least cross-validation error.

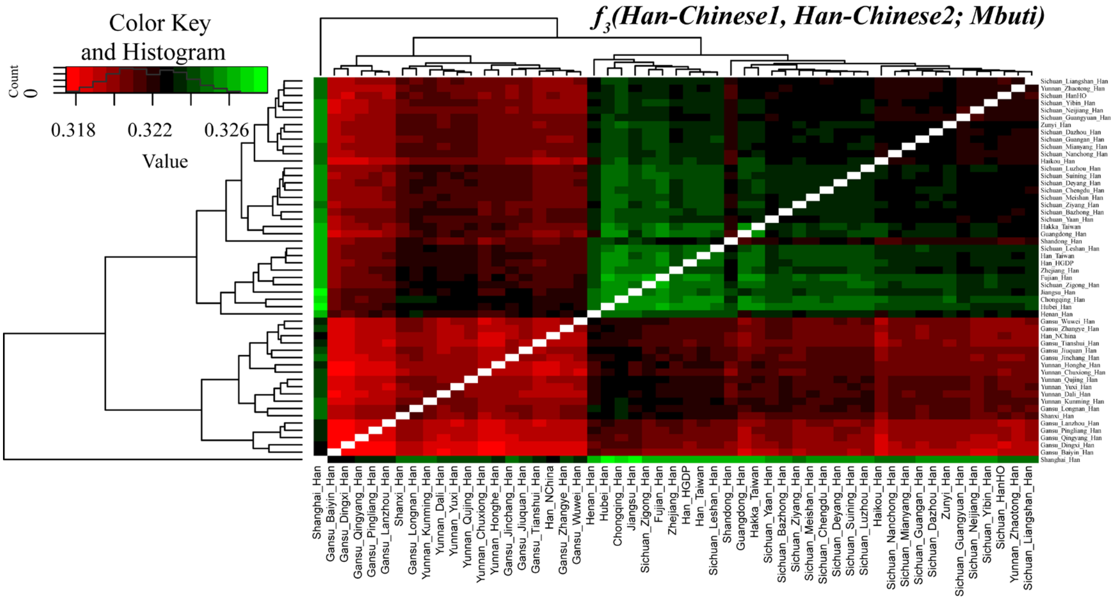

Figure S7. Pairwise shared genetic drift among Han Chinese individuals.

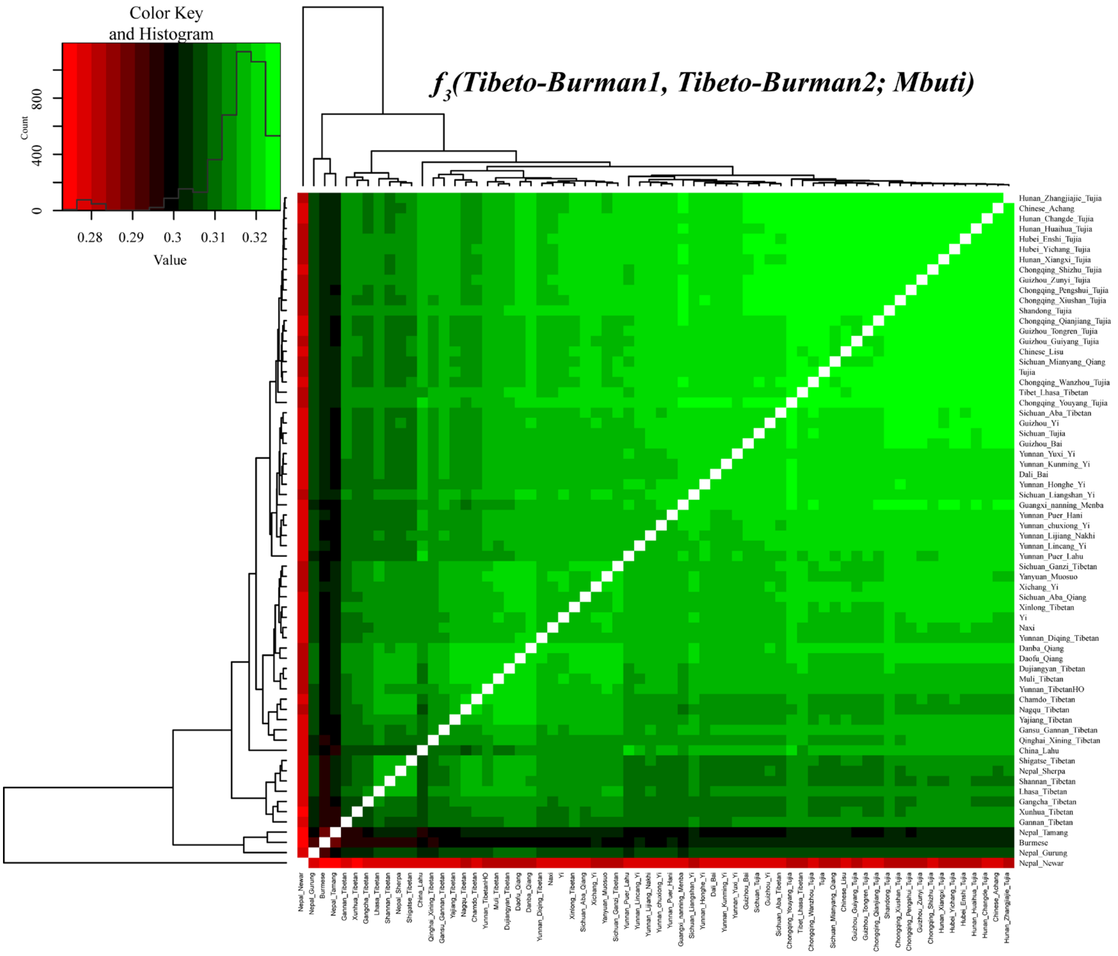

Figure S8. Heatmap of pairwise shared genetic affinity among Tibeto-Burman groups.

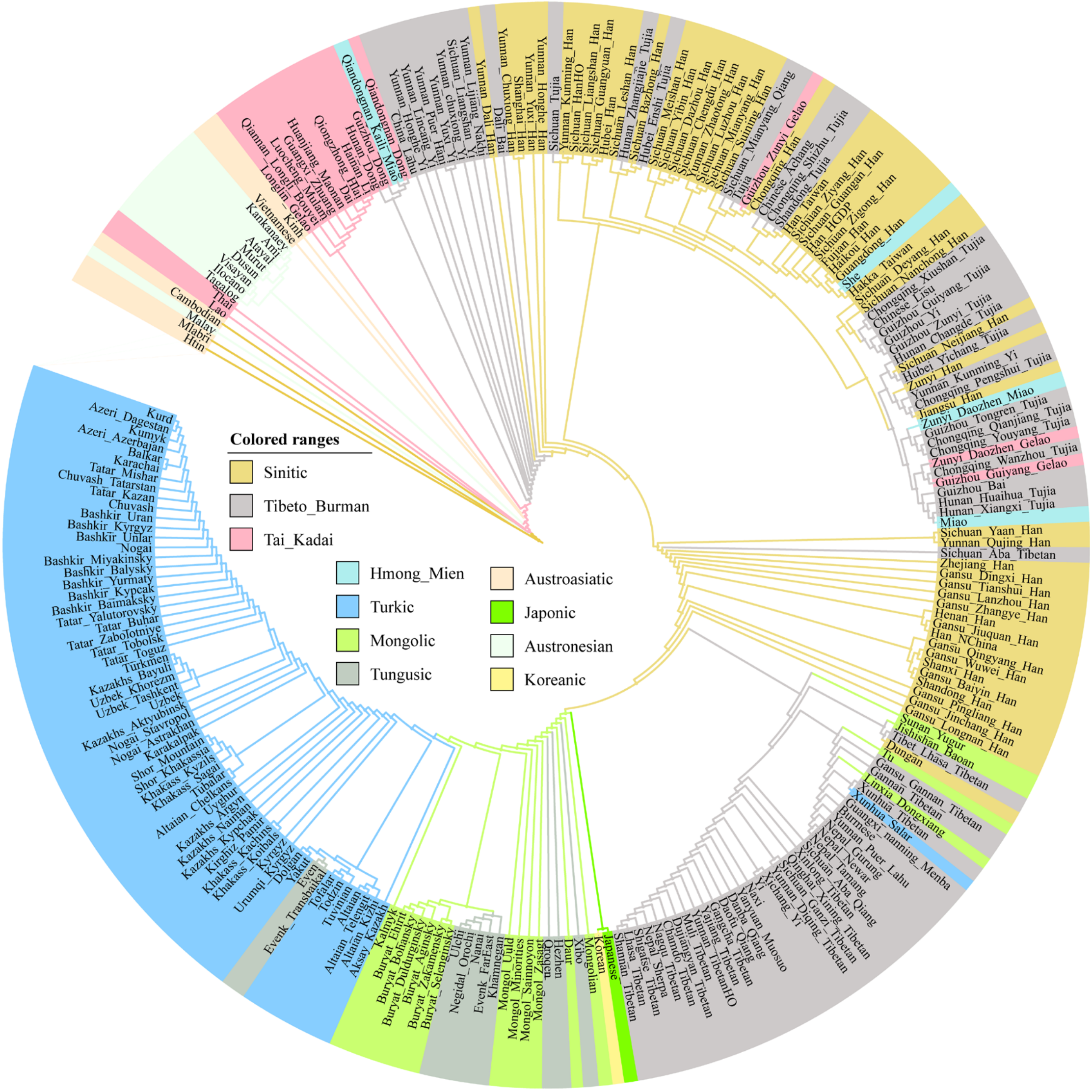

Figure S9. Phylogenetic relationships among 242 populations constructed from the HO_Affy dataset using pairwise Fst genetic distances.

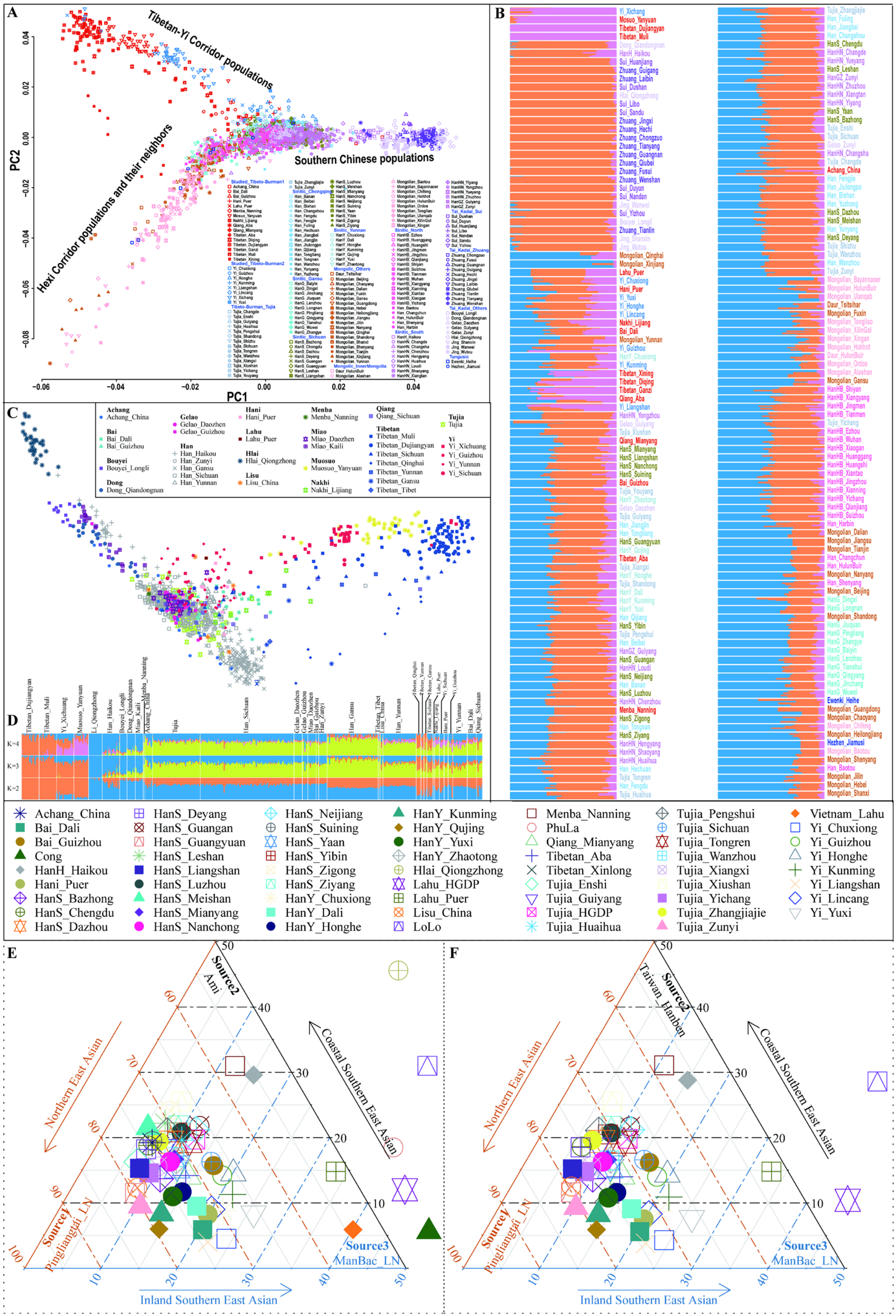

Figure S10. Genetic structure of Sino-Tibetan groups along the Tibetan-Yi and Hexi corridors inferred from the Affymetrix dataset. A, Population structure of 2,298 individuals from 188 East Asian populations. **B**, Admixture landscape of the same 188 populations, following the population groupings and color scheme used in Fig. S10A. Population stratification of newly genotyped Sino-Tibetan-speaking and southern East Asian populations inferred from PCA (**C**) and model-based ADMIXTURE analysis (**D**). Ancestry composition of Tibetan-Yi Corridor populations modeled as three-way admixtures: Pingliangtai_LN–Ami–ManBac_LN (**E**) and Pingliangtai_LN–Hanben–ManBac_LN (**F**).

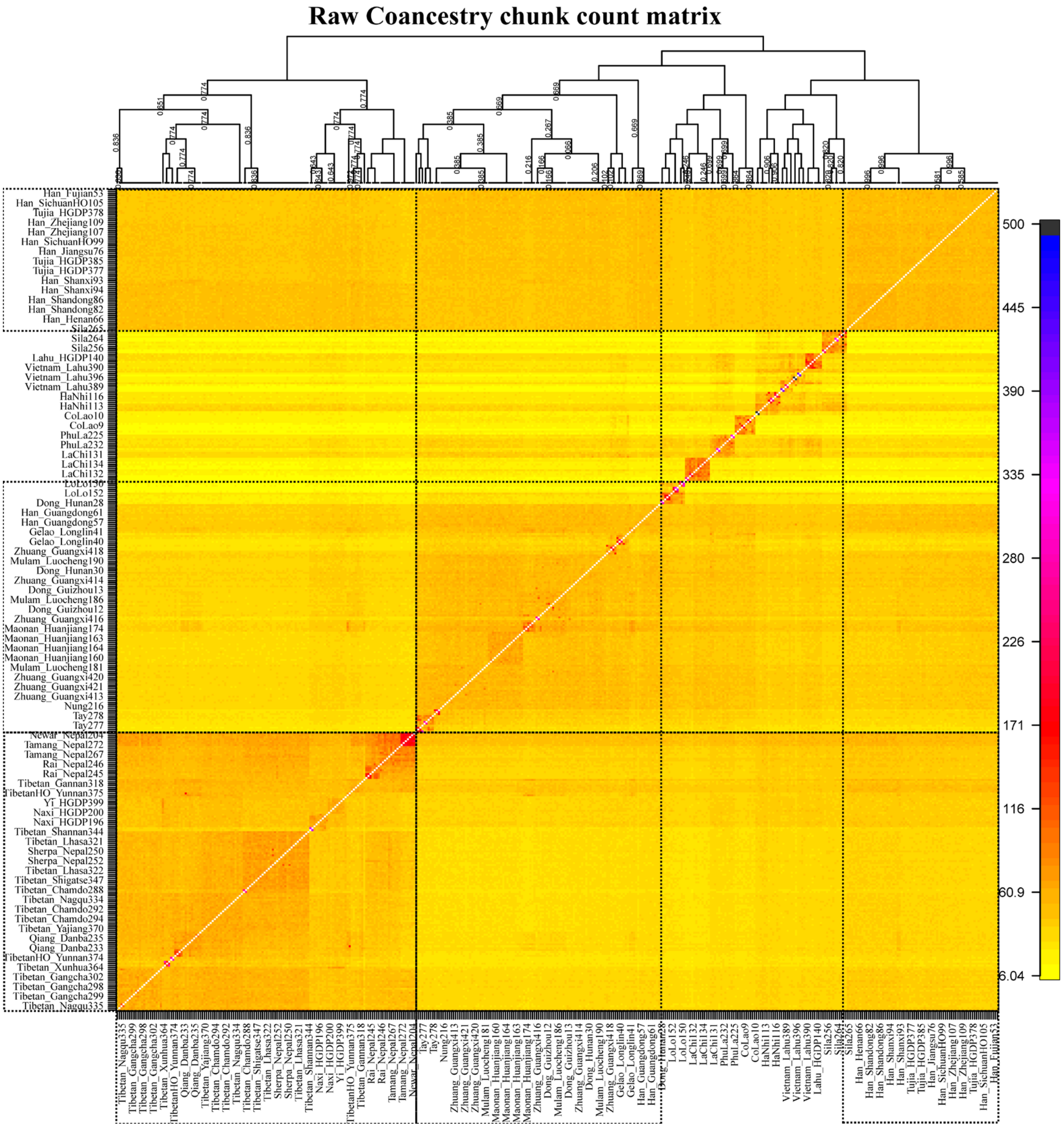

Figure S11. Coancestry heatmap for the linked model showing the unlinked model among 50 Sino-Tibetan-speaking populations, noting that the linked heatmap is slightly asymmetric.

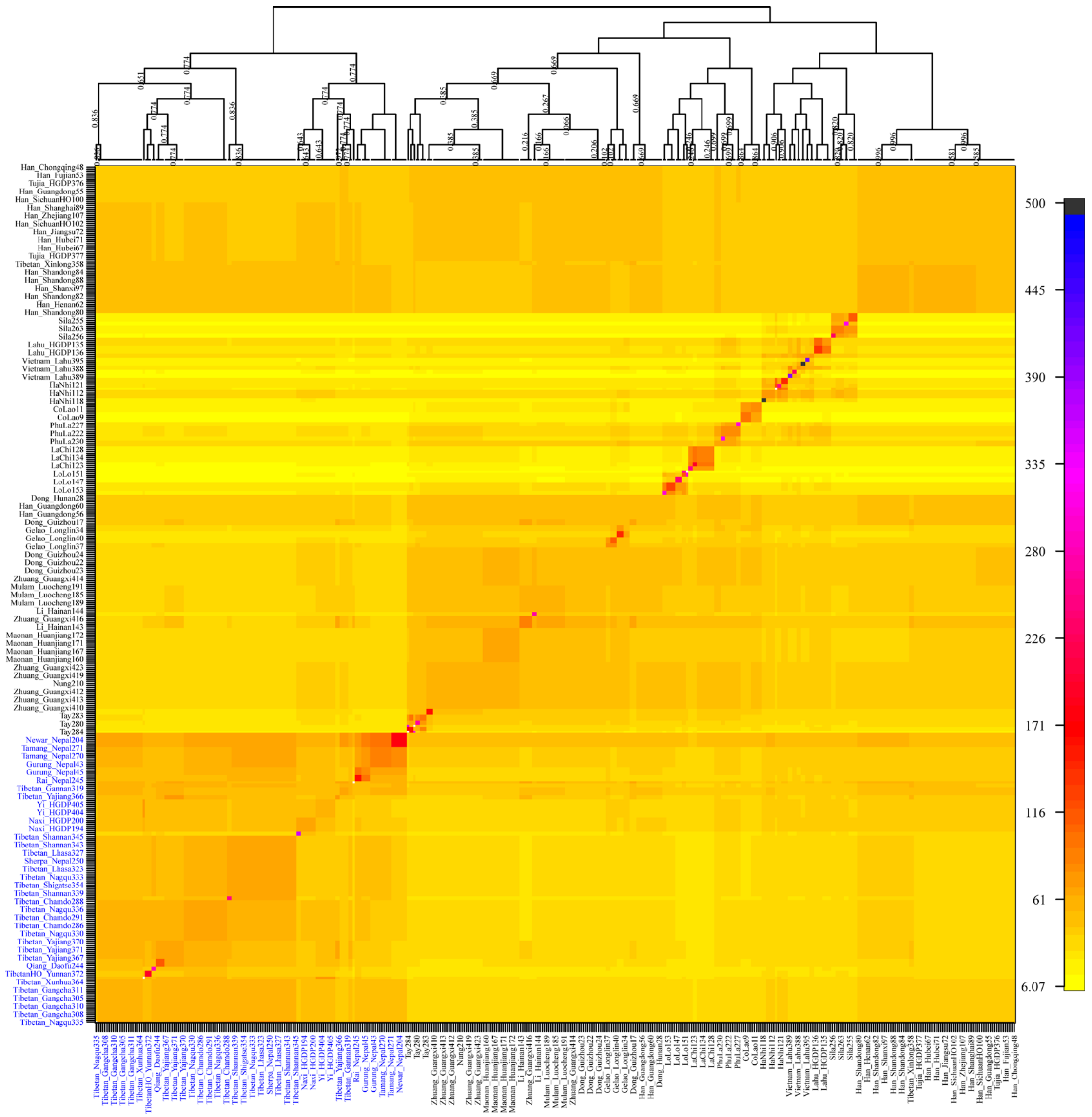

Figure S12. Population average coancestry heatmaps for the linked model showing the unlinked model among 50 Sino-Tibetan-speaking populations, noting that the linked heatmap is slightly asymmetric.

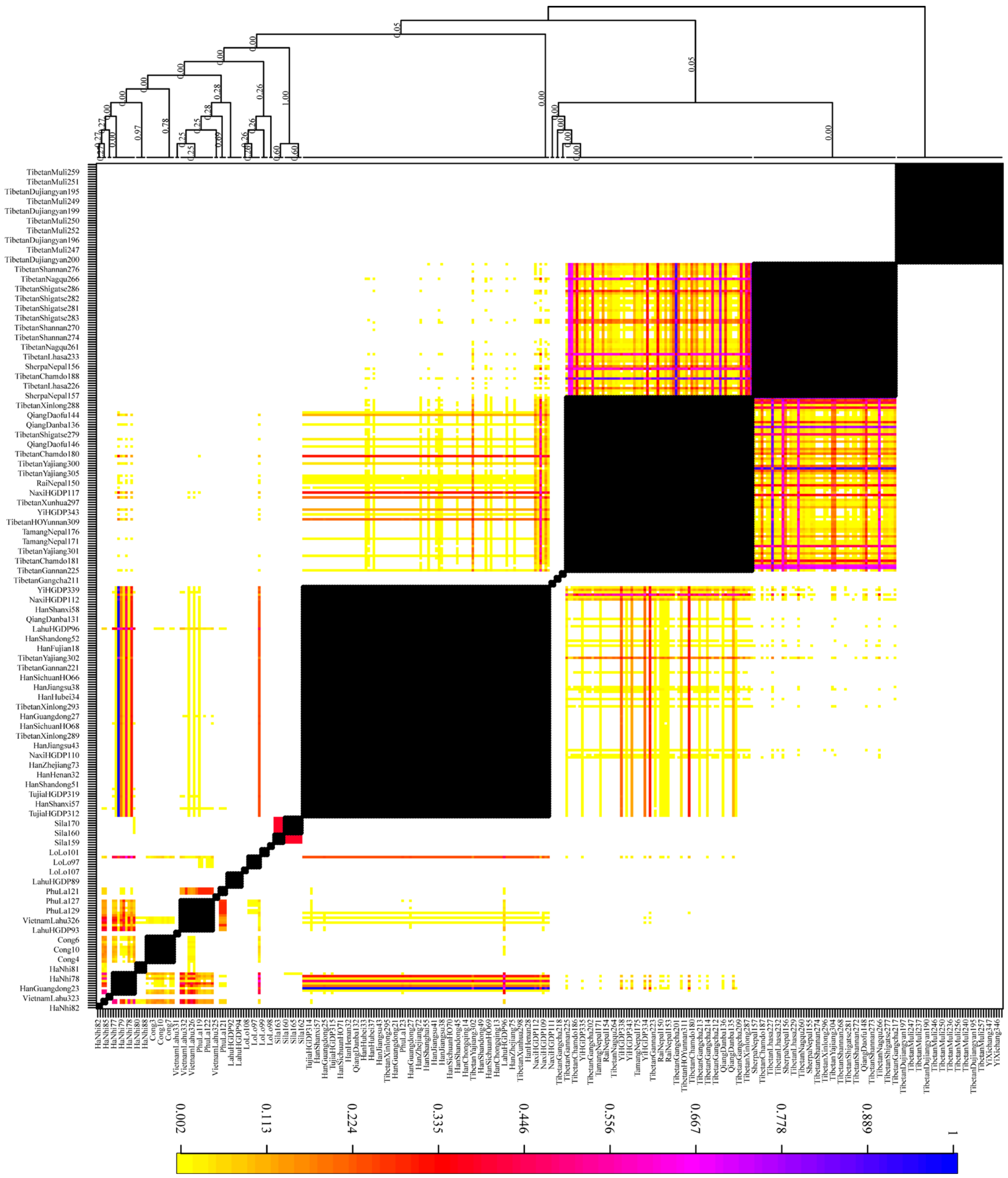

Figure S13. Results of population assignment among 50 Sino-Tibetan-speaking populations. Pairwise coincidence matrix output by fineSTRUCTURE using chunk counts calculated using the linked model. The coloring represents the posterior coincidence probability (which does not drop below 97%), and the dots represent the maximum a posteriori (MAP) probability state.

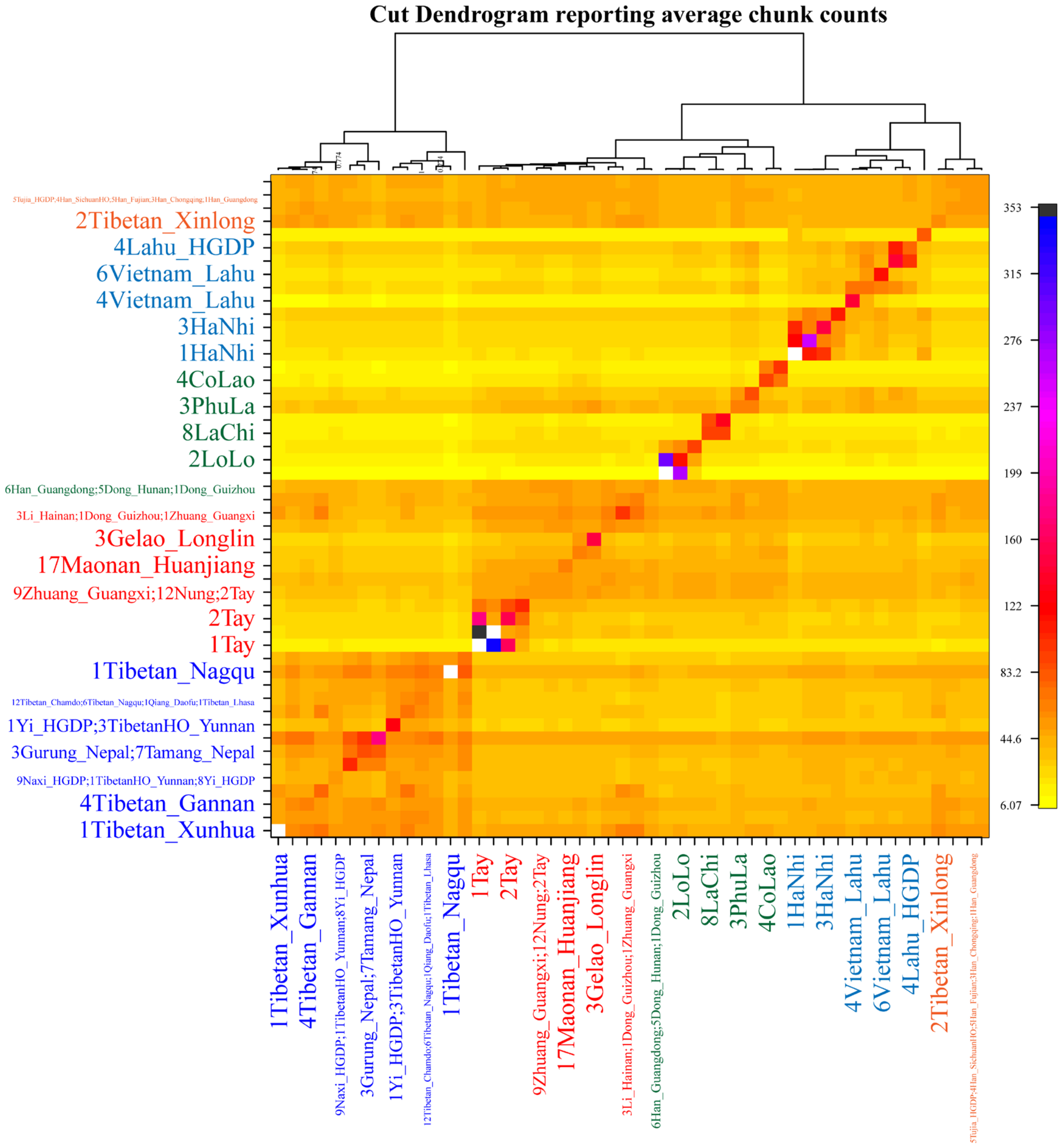

Figure S14. Cut dendrogram showing average chunk counts among 50 Sino-Tibetan-speaking populations.

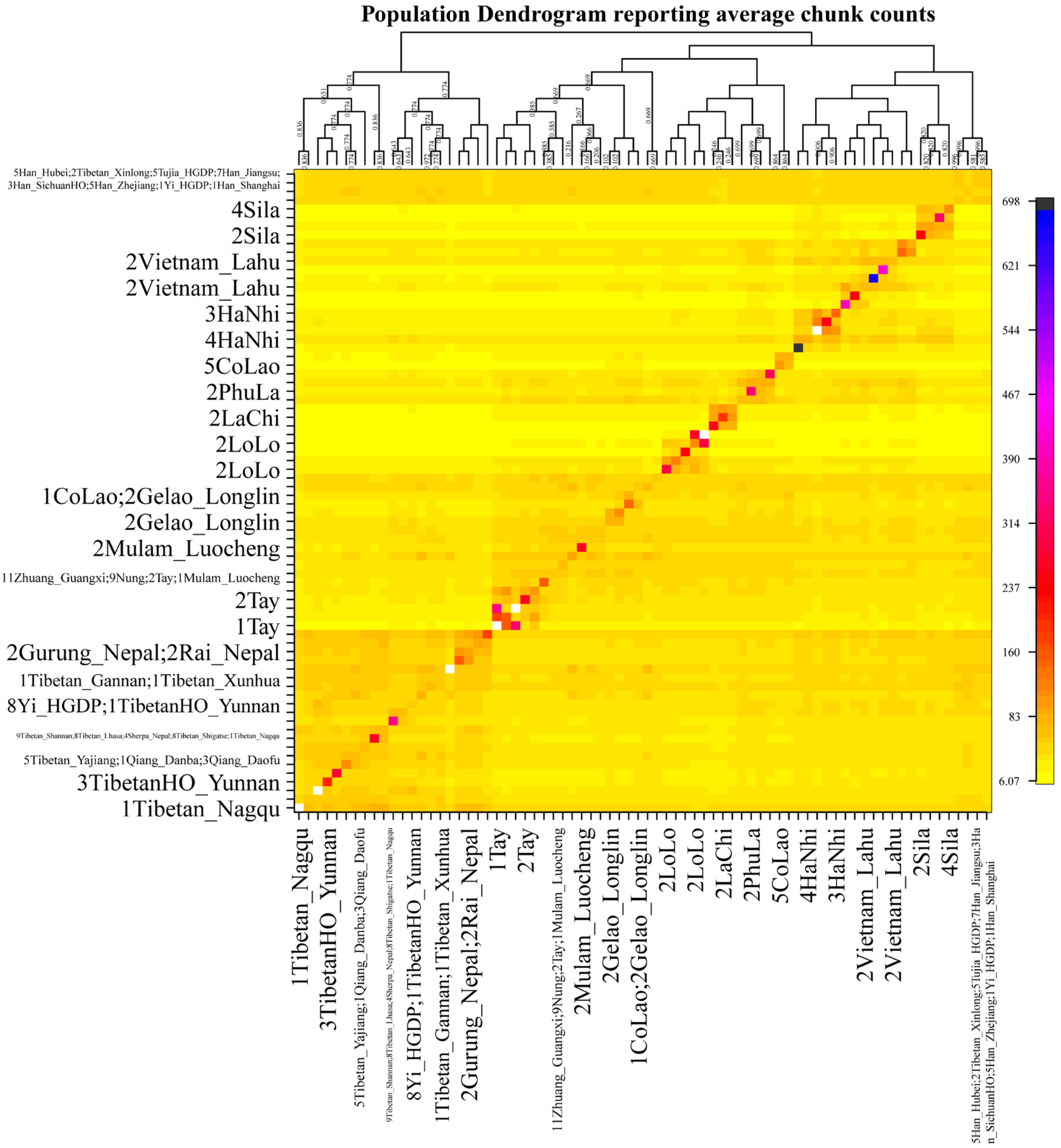

Figure S15. Population dendrogram showing average chunk counts among 50 Sino-Tibetan-speaking populations.

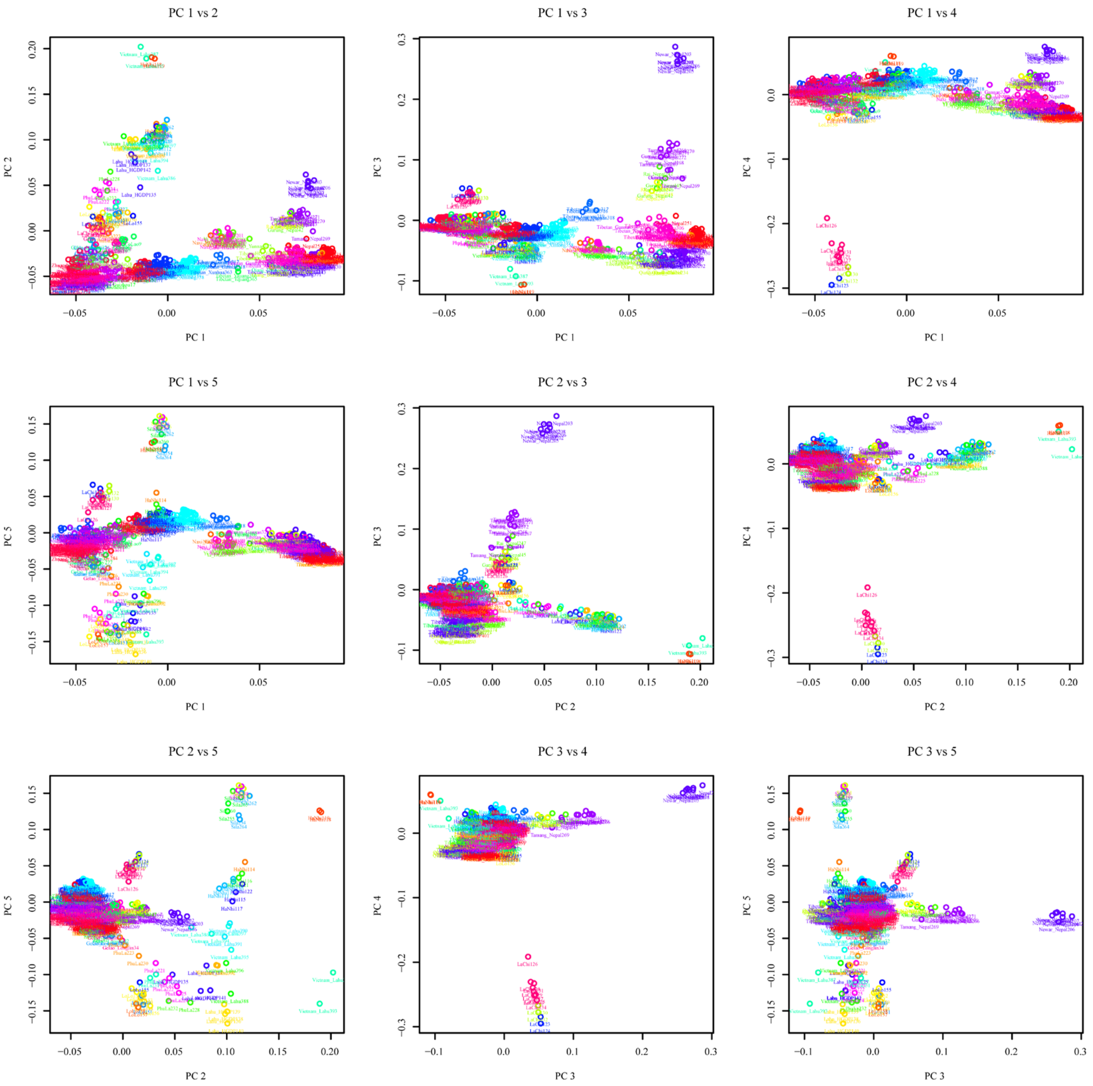

Figure S16. Principal component analysis using the coancestry matrix among 50 Sino-Tibetan-speaking populations.

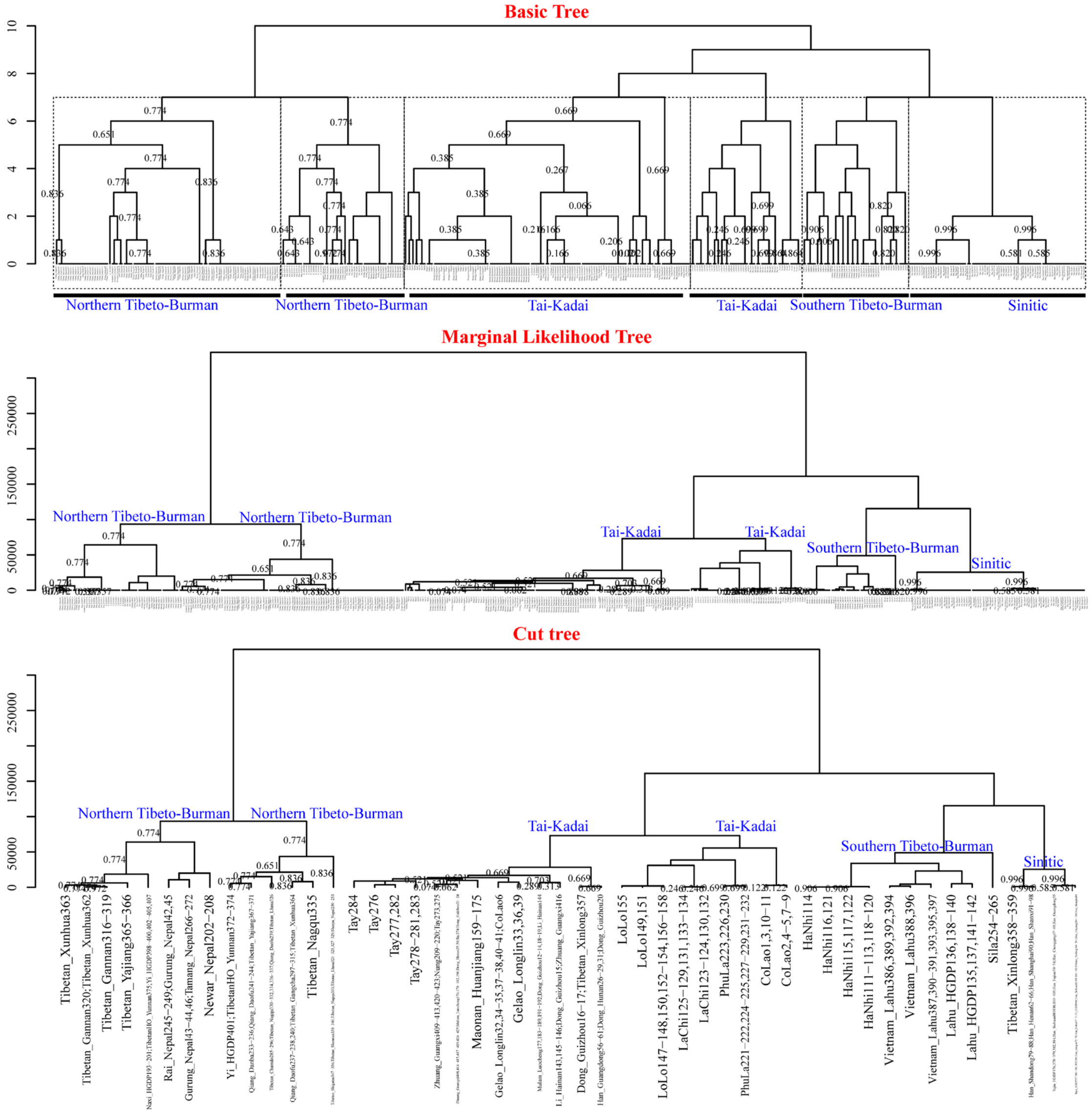

Figure S17. Model-based likelihood of the coancestry matrix among 50 Sino-Tibetan-speaking populations.

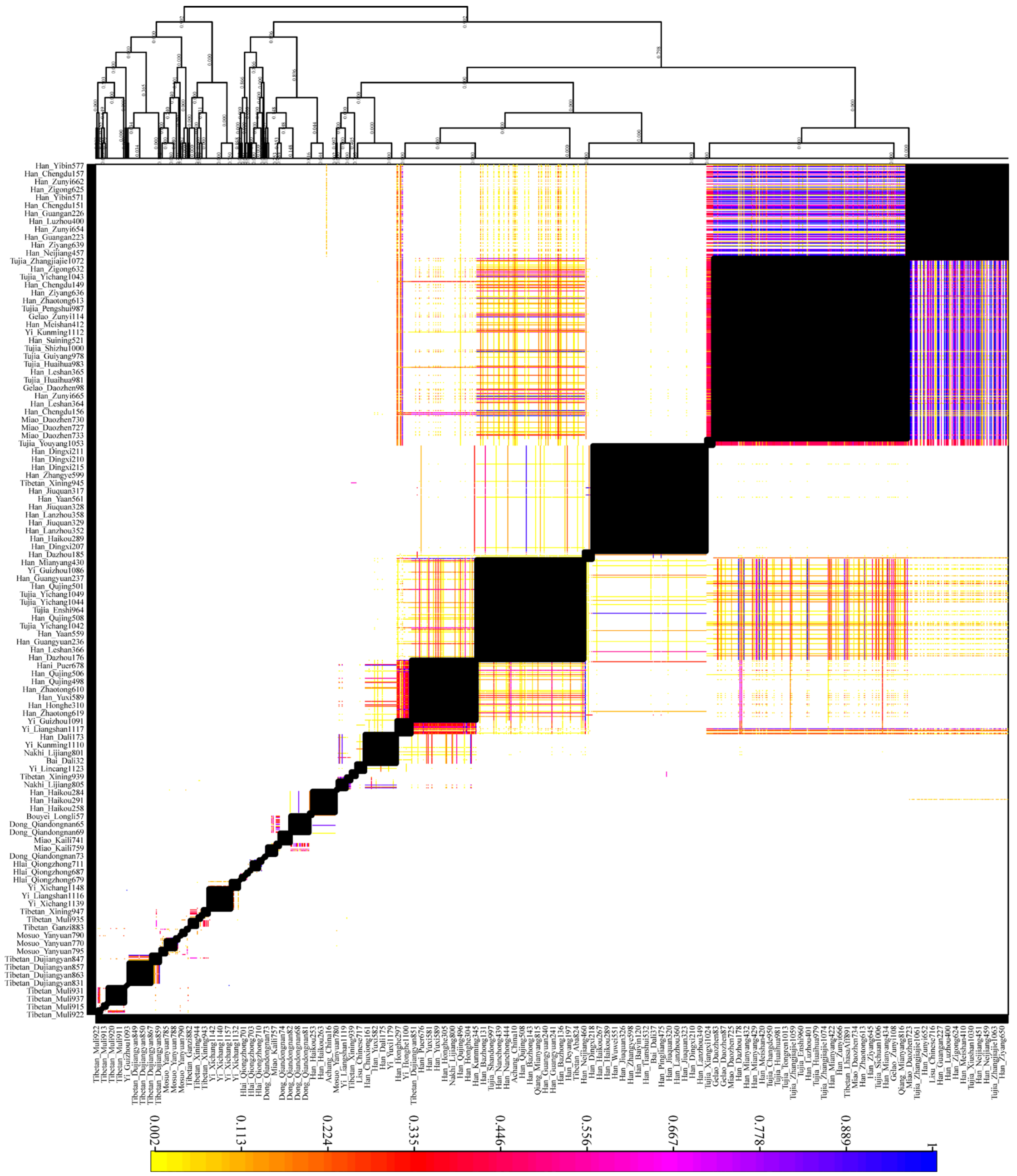

Figure S18. Results of population assignment among 41 Sino-Tibetan-speaking populations. Pairwise coincidence matrix output by fineSTRUCTURE using chunk counts calculated using the linked model. The coloring represents the posterior coincidence probability (which does not drop below 97%), and the dots represent the maximum a posteriori (MAP) probability state.

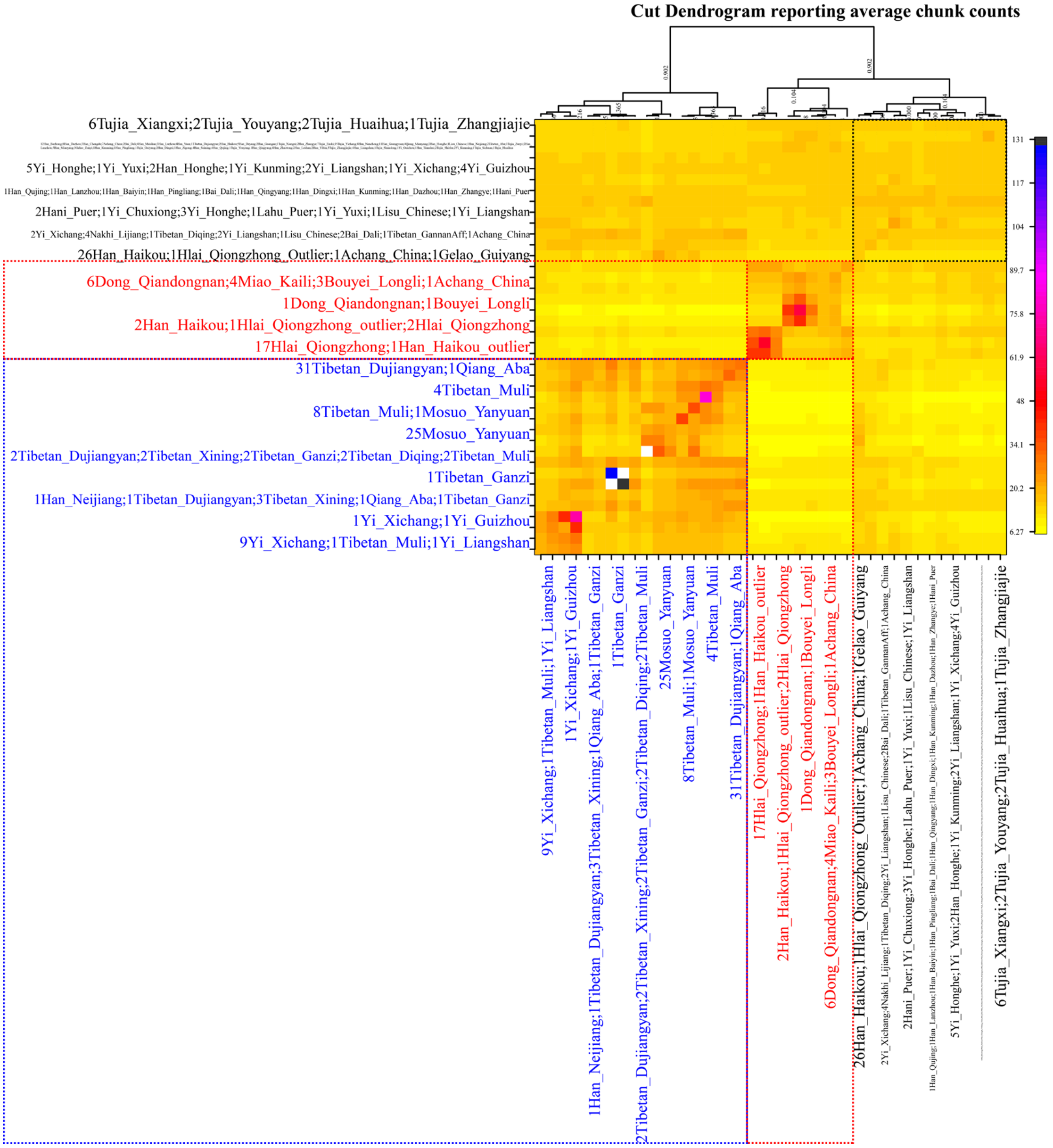

Figure S19. Cut dendrogram showing average chunk counts among 41 Sino-Tibetan-speaking populations.

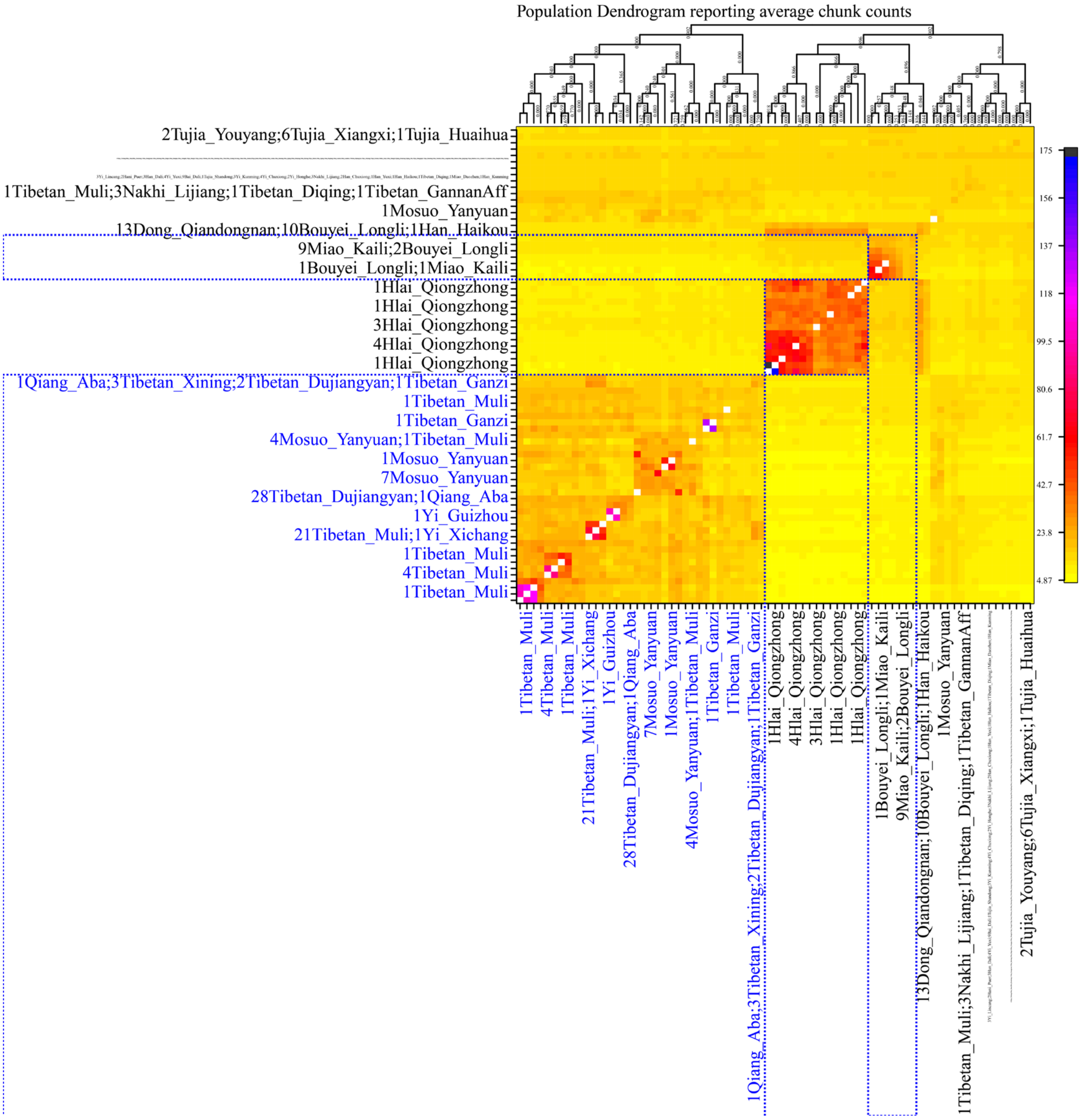

Figure S20. Population dendrogram showing average chunk counts among 41 Sino-Tibetan-speaking populations.

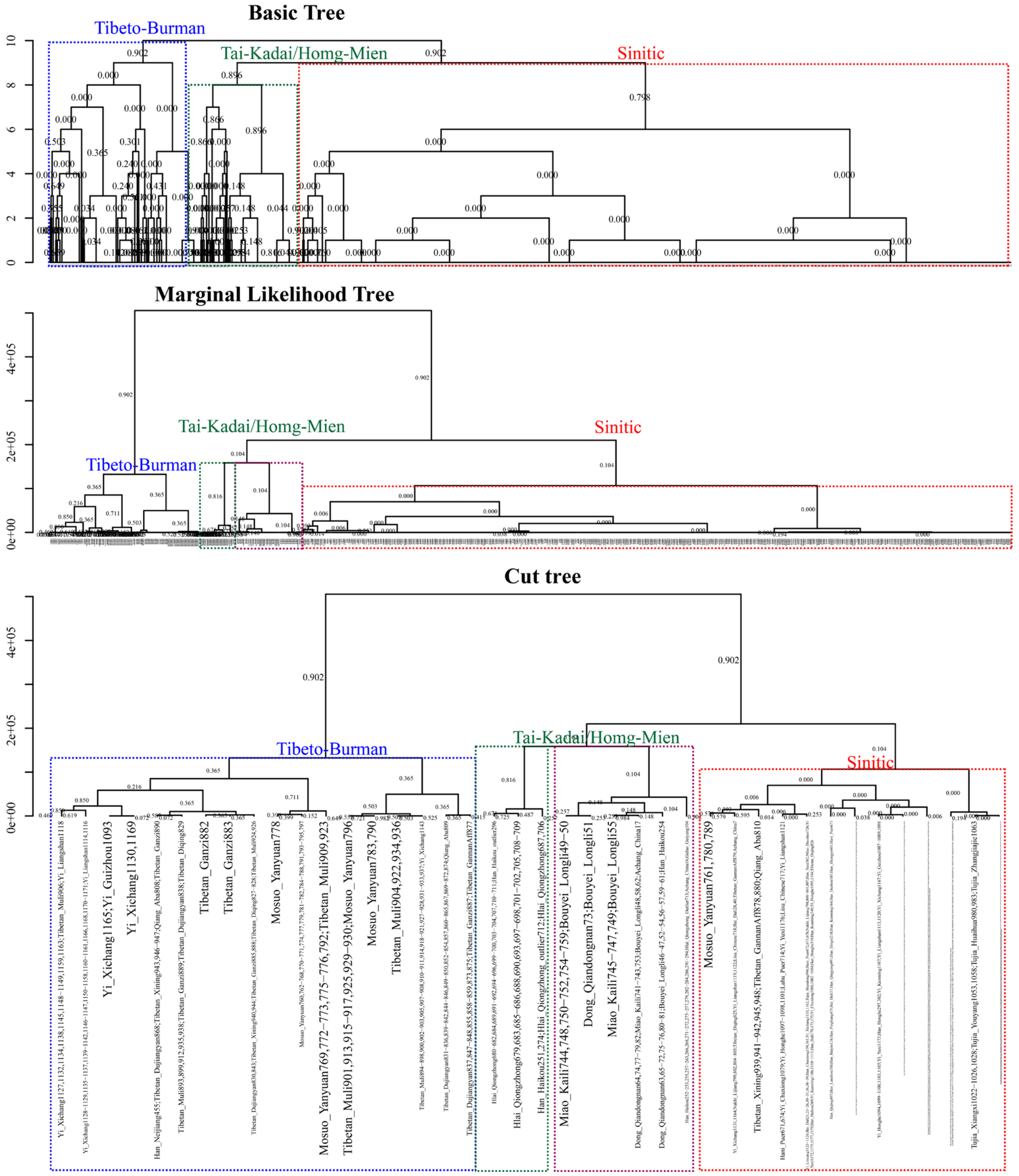

Figure S21. Phylogenetic tree reconstructed based on the shared ancestral fragment length among 41 Sino-Tibetan-speaking populations.

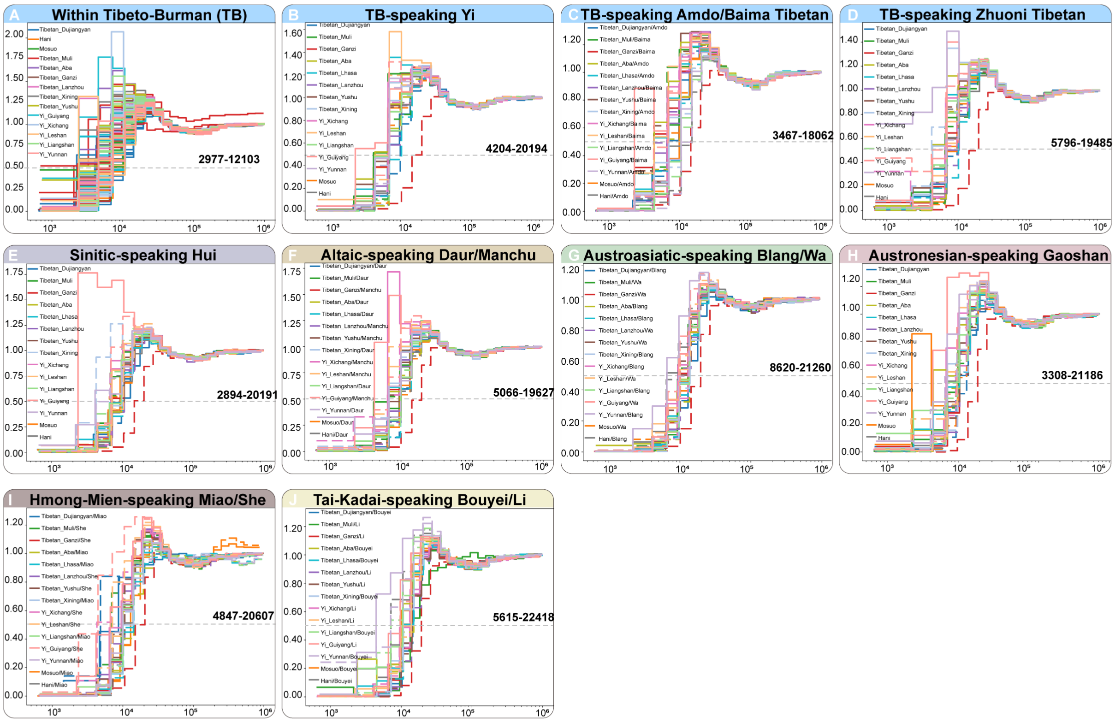

FIG. 22. Complex demographic histories of Tibeto-Burman-speaking populations in the Tibetan-Yi and Hexi corridors. (*A*) Intragroup divergence times among the newly sequenced Tibeto-Burman-speaking populations. (*B–D*) Pairwise divergence times between focal Tibeto-Burman groups and reference populations: southern Tibeto-Burman-speaking Anha Yi from Sichuan (*B*), Amdo Tibetan and Baima Tibetan (*C*), and northern Tibeto-Burman-speaking Zhuoni Tibetan from Gansu (*D*); (*E*) Divergence times between focal Tibeto-Burman groups and Sichuan Hui; (*F–J*) Divergence times between newly genotyped Tibeto-Burman groups and linguistically diverse non-Sino-Tibetan speakers: Altaic-speaking Daur and Manchu from Inner Mongolia (*F*); Austroasiatic-speaking Blang and Wa from Yunnan (*G*); Austronesian-speaking Gaoshan from Fujian and Taiwan (*H*); Hmong-Mien-speaking Sichuan Miao and Fujian She (*I*); and Tai-Kadai-speaking Guizhou Bouyei and Hainan Li (*J*). We removed lines indicating overly recent divergence times from the main graph, as these likely reflect close genetic relationships between the study and reference populations.

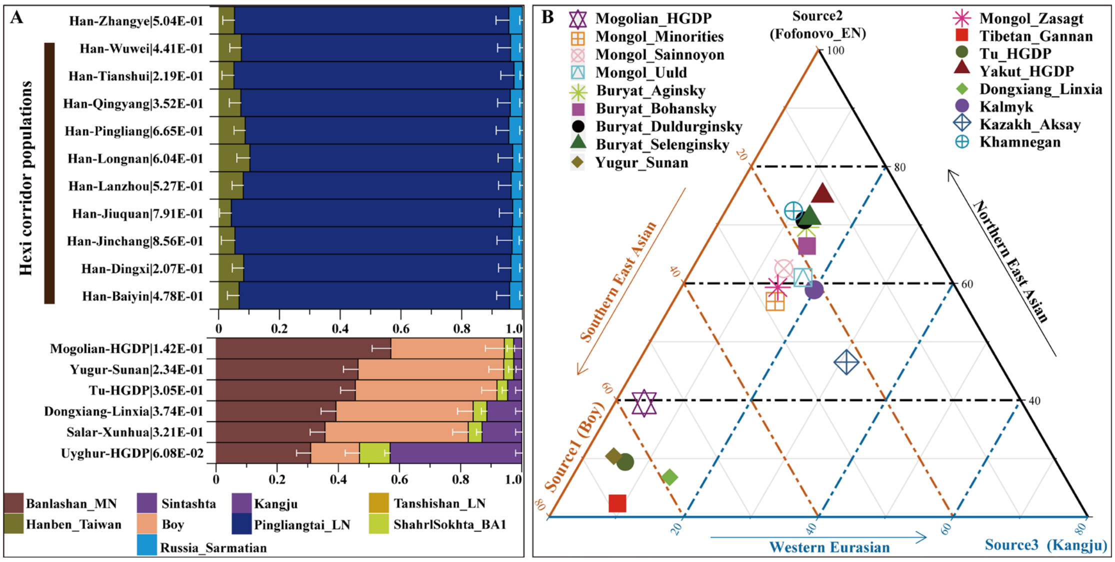

Figure S23. Admixture profiles of modern populations from the Hexi Corridor and adjacent regions. A, Three-way admixture models for northwestern Han Chinese, inferred from the merged 1240K dataset. The models include Neolithic northern East Asians (Pingliangtai_LN), ancient southern East Asians (Hanben), and Sarmatian pastoralists as representatives of Western Eurasian ancestry. The lower panel presents four-way admixture models incorporating two East Asian and two Western Eurasian sources, highlighting the intricate ancestral composition of northwestern East Asian populations. **B**,

Three-way admixture models for ethnic minorities in Northwest China, incorporating one Western Eurasian and two East Asian sources, based on the HO_Affy dataset.
